# The association between genetic polymorphisms in ABCG2 and SLC2A9 and urate: an updated systematic review and meta-analysis

**DOI:** 10.1186/s12881-020-01147-2

**Published:** 2020-10-21

**Authors:** Thitiya Lukkunaprasit, Sasivimol Rattanasiri, Saowalak Turongkaravee, Naravut Suvannang, Atiporn Ingsathit, John Attia, Ammarin Thakkinstian

**Affiliations:** 1 Department of Clinical Epidemiology and Biostatistics, Faculty of Medicine, Ramathibodi Hospital, Mahidol University, 270 Rama VI Rd., Ratchathewi, Bangkok, 10400 Thailand; 2 Department of Pharmacology, College of Pharmacy, Rangsit University, Pathum Thani, Thailand; 3 Social and Administrative Pharmacy Excellence Research (SAPER) Unit, Department of Pharmacy, Faculty of Pharmacy, Mahidol University, Bangkok, Thailand; 4 Centre for Clincial Epidemiology and Biostatistics, School of Medicine and Public Health, Faculty of Health and Medicine, University of Newcastle, and Hunter Medical Research Institute, Newcastle, NSW Australia

**Keywords:** *ABCG2*, Gout, Hyperuricemia, Meta-analysis, Single nucleotide polymorphism, *SLC2A9*, Urate

## Abstract

**Background:**

Replication studies showed conflicting effects of *ABCG2* and *SLC2A9* polymorphisms on gout and serum urate. This meta-analysis therefore aimed to pool their effects across studies.

**Methods:**

Studies were located from MEDLINE and Scopus from inception to 17th June 2018. Observational studies in adults with any polymorphism in *ABCG2* or *SLC2A9*, and outcome including gout, hyperuricemia, and serum urate were included for pooling. Data extractions were performed by two independent reviewers. Genotype effects were pooled stratified by ethnicity using a mixed-effect logistic model and a multivariate meta-analysis for dichotomous and continuous outcomes.

**Results:**

Fifty-two studies were included in the analysis. For *ABCG2* polymorphisms, mainly studied in Asians, carrying 1–2 minor-allele-genotypes of rs2231142 and rs72552713 were respectively about 2.1–4.5 and 2.5–3.9 times higher odds of gout than non-minor-allele-genotypes. The two rs2231142-risk-genotypes also had higher serum urate about 11–18 μmol/l. Conversely, carrying 1–2 minor alleles of rs2231137 was about 36–57% significantly lower odds of gout. For *SLC2A9* polymorphisms, mainly studied in Caucasians, carrying 1–2 minor alleles of rs1014290, rs6449213, rs6855911, and rs7442295 were about 25–43%, 31–62%, 33–64%, and 35–65% significantly lower odds of gout than non-minor-allele-genotypes. In addition, 1–2 minor-allele-genotypes of the latter three polymorphisms had significantly lower serum urate about 20–49, 21–51, and 18–54 μmol/l than non-minor-allele-genotypes.

**Conclusions:**

Our findings should be useful in identifying patients at risk for gout and high serum urate and these polymorphisms may be useful in personalized risk scores.

**Trial registration:**

**PROSPERO registration number:** CRD42018105275.

**Supplementary information:**

The online version contains supplementary material available at 10.1186/s12881-020-01147-2.

## Introduction

Hyperuricemia, defined as serum urate > 7 mg/dl (or 416.4 μmol/l) [1], can lead to gout [1] and increased risk of renal disease, diabetes, hypertension, and cardiovascular disease [2]. Genome-wide association studies (GWAS) have shown that many single nucleotide polymorphisms (SNPs) of ATP-binding cassette sub-family G member 2 gene (*ABCG2*) and the solute carrier family 2 member 9 (*SLC2A9)* are associated with serum urate and gout [3–6]. Both *ABCG2* and *SLC2A9* are located on chromosome 4 [7]. Many more individual studies have replicated the findings of GWASs and these were summarized in systematic reviews of the effects of *ABCG2* [8–13] and *SLC2A9* [12, 14–16] polymorphisms on gout. The most updated review of *ABCG2* [12] included 12 individual studies, indicating that carrying the A allele of rs2231142 increased the risk of gout by about 1.8–2.3 times relative to the C allele in Asian and non-Asian populations. The most recent review of *SLC2A9* [15] included 13 studies indicating that rs6449213 (C versus T), rs16890979 (T versus C) and rs1014290 (C versus T) significantly decreased the risk of gout whereas rs3733591 increased the risk of gout only in Asian and Maori populations and Solomon Islanders.

There have been 12 studies of *ABCG2*-rs2231142 on gout published after these reviews and most reviews did not pool genotype effects, which could lead to suggest mode of gene effects. In addition, the effects of *ABCG2* and *SLC2A9* polymorphisms on serum urate have never been systematically reviewed. Therefore, an updated systematic review and meta-analysis regarding the influence of SNPs in *ABCG2* and *SLC2A9* on gout, hyperuricemia and serum urate is needed.

## Methods

### Search terms and strategies

Studies were located from MEDLINE (via PubMed search engine) and Scopus databases from inception to 17th June 2018. The search terms and strategies were constructed based on study genes (i.e., *ABCG2* and *SLC2A9* genes) and outcomes (i.e., gout, hyperuricemia and serum urate); see more details in Additional file 1. This study was registered in PROSPERO number CRD42018105275.

### Inclusion/exclusion criteria

Screening of titles and abstracts were performed by TL and randomly checked by SR. Any type of observational study was selected as follows: studies in adults, with any polymorphism in *ABCG2* or *SLC2A9*, and any outcome including gout, hyperuricemia, and serum urate. Studies with insufficient data and unavailable full-texts were excluded.

### Data extraction

Two of 3 authors (i.e., TL, SR, ST, and NS) independently extracted data. Extracted information included study characteristics (study setting, study design), participants (ethnicity, age, gender, body mass index (BMI), estimated glomerular filtration rate (eGFR), co-morbidity, medications, and alcohol consumption), genes (*ABCG2/SLC2A9*/both, allele/genotype, and frequency/summary data), and outcomes (gout and its diagnostic criteria, hyperuricemia and its definition, and serum urate). In addition, data for pooling (frequency data for allele/genotype and outcome, and mean and standard deviation (SD) of urate by allele/genotype) were also extracted. Missing data was requested from authors. Any disagreement was resolved by discussion and consensus.

### Risk of bias assessment

Risk of bias was independently assessed by two reviewers (TL and SR) using a risk-of-bias tool [17] which consisted of 4 domains, i.e., information bias, confounding bias, selective reporting, and Hardy-Weinberg equilibrium (HWE). Each question was answered as yes, no, and unclear representing low/no, possible/high, and unclear risk of bias due to insufficient information, respectively. Any disagreement was resolved by discussion and consensus with the team.

### Study genes and outcome of interest

Data for the following polymorphisms were collected: *ABCG2* gene (rs2231142 C > A, rs72552713 C > T and rs2231137 C > A) and *SLC2A9* gene (rs1014290 G > A, rs3733591 G > A, rs6449213 G > A, rs6855911 G > A, rs7442295 G > A, rs12510549 G > A, rs16890979 G > A, and rs734553 T > G). Outcomes of interest were gout, hyperuricemia, and serum urate defined according to original studies.

### Statistical analysis

Statistical analysis was performed using methods previously described [18]. HWE was checked using an exact test and only studies which complied with HWE were considered in the analysis. Prevalence of the minor allele of each SNP was pooled, and stratified by ethnicity. Gene effects were assessed as follows:

#### Per-allele approach

For hyperuricemia and gout, allele effect (i.e., odds ratio (OR)) for minor allele a versus A along with 95% confidence interval (CI) was estimated. Mean difference (MD) of urate, in μmol/l, for a versus A was estimated. Heterogeneity was explored using Cochrane’s Q test and I^2^. A random-effect model was used for pooling ORs if heterogeneity was present (*P*-value < 0.10 or I^2^ ≥ 25%), otherwise a fixed-effect model was applied. Sources of heterogeneity (e.g. age, gender, co-morbidity, etc.) were explored using meta-regression if the data were available, and subgroup analyses were then performed accordingly.

#### Per-genotype approach

ORs (i.e., OR_1_ (aa versus AA) and OR_2_ (Aa versus AA)) and mean differences (MDs) (MD_1_ (aa versus AA) and MD_2_ (Aa versus AA)) were estimated for dichotomous and continuous outcomes, respectively. Heterogeneity was explored as above. Aggregated genotype and outcome data were expanded to individual patient data (IPD). A one-stage approach with a mixed-effect logistic regression was applied by fitting genotypes (i.e., aa versus AA and Aa versus AA) on outcome, and pooled ORs were estimated. For serum urate, a multivariate (mv) meta-analysis was applied to pool MD_1_ and MD_2_ across studies. Mode of gene effects (i.e., dominant, recessive, additive) was then determined by estimating lambda (λ) (i.e., logOR_2_/logOR_1_ or MD_2_/MD_1_) using model-free Bayesian approach [19].

Sensitivity analysis was performed by including studies with HWE disequilibrium. Publication bias was assessed using funnel plots and Egger’s tests. If any of these suggested asymmetry, a contour enhanced-funnel was constructed to determine whether asymmetry was caused by publication bias or heterogeneity.

STATA software version 15.1 and WinBUGS version 1.4.3 were used for analyses. The level of significance was < 0.05 except for the heterogeneity test, in which < 0.10 was used.

## Results

### Identifying studies

**Fig. 1:**
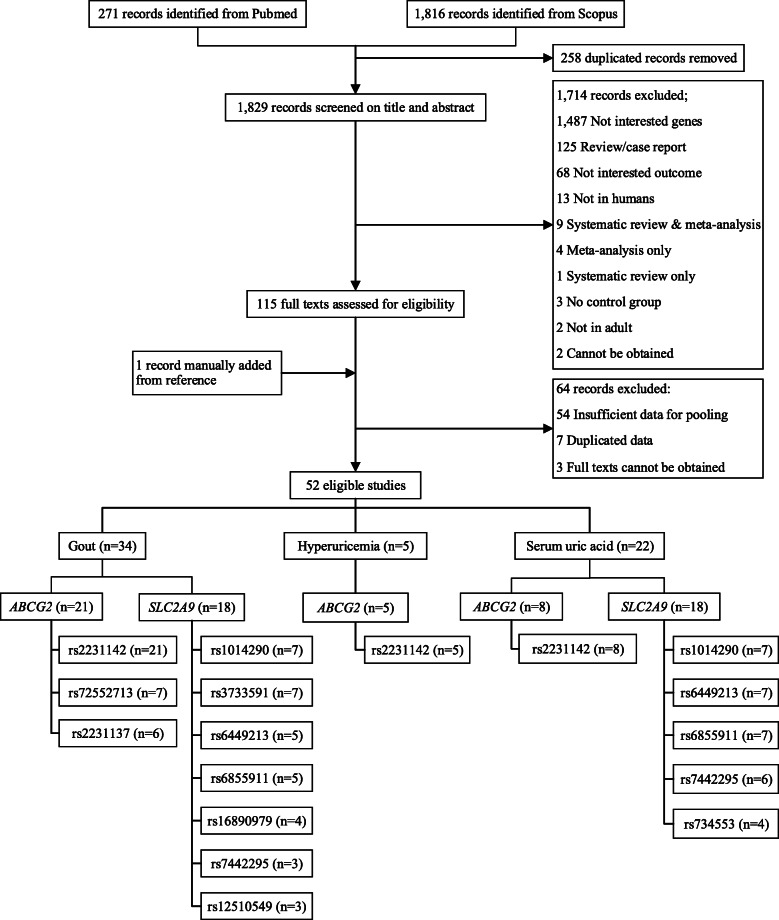
Flow for identifying and selecting studies. *ABCG2*, ATP-binding cassette sub-family G member 2; *SLC2A*9, solute carrier family 2 member 9

Among 2087 identified studies, 52 studies were eligible, which included one study manually added from the reference, see Fig. 1. Among them, 34, 5, and 22 studies had outcomes of gout, hyperuricemia, and serum urate, respectively.

The characteristics of 52 studies (i.e., 30 *ABCG2* studies [20–49] and 32 *SLC2A9* studies [24–26, 28, 31, 33, 39, 40, 43, 45, 50–71]) with a total of 63 sub-studies are described in Additional file 2. Most studies were cohorts (50%), and split roughly evenly between Asians (46%) and Caucasians (42%). The mean ages and BMIs ranged from 37 to 66 years and from 22.6 to 34.6 kg/m^2^, respectively. Of 34 studies on gout, most studies (68%) used the criteria of American College of Rheumatology [72] for diagnosis. All studies used the cut-off of 416.4 μmol/l (or 7 mg/dl) for defining hyperuricemia.

### Risk of bias assessment

The risk of bias was low for population stratification and selective outcome report (see Additional file 3 A-B). Twenty sub-studies were at high risk of bias in ascertainment of genotyping because they did not clearly describe the methods they used. Furthermore, 17 sub-studies had high risk of bias in ascertainment of outcome mostly due to no information clearly mentioned.

### Gout

Among 34 studies, 3 SNPs in *ABCG2* (i.e., rs2231142, rs72552713, and rs2231137) and 7 SNPs in *SLC2A9* (i.e., rs1014290, rs3733591, rs6449213, rs16890979, rs6855911, rs7442295, and rs12510549) had sufficient data for pooling as described in Additional file 4.1. Minor allele prevalences of these polymorphisms were pooled, see Additional file 4.2.

**Fig. 2:**
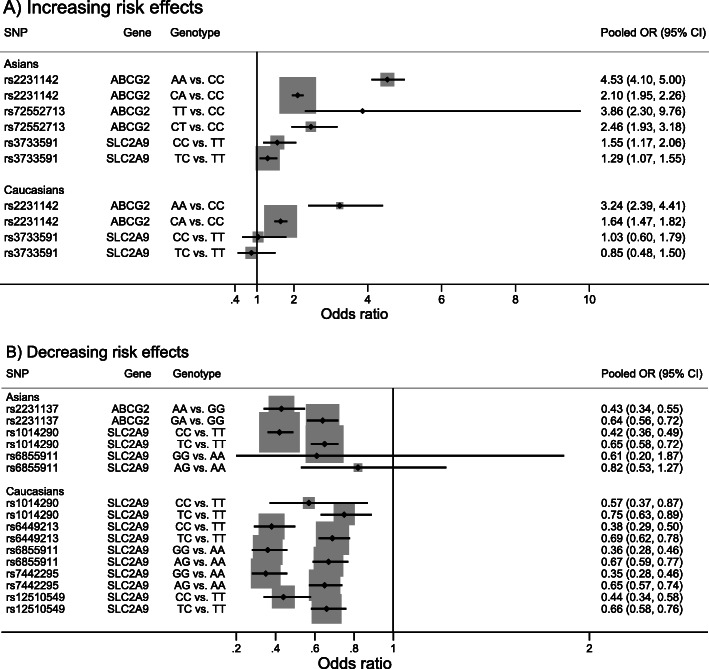
Summary of pooled genotype effects on gout. **a** Increasing risk effects **b** Decreasing risk effects. *ABCG2*, ATP-binding cassette sub-family G member 2; CI, confidence interval; OR, odds ratio; *SLC2A9*, solute carrier family 2 member 9; SNP, single nucleotide polymorphism

Genotype effects of *ABCG2* polymorphisms (i.e., rs2231142 (*N* = 21), rs72552713.

(*N* = 7), and rs2231137 (*N* = 6)) were estimated stratifying by Asians and Caucasians, see Fig. 2, Table 1, and Additional file 4.1. In Asian populations, carrying the homozygous minor and heterozygous genotypes of rs2231142 and rs72552713 were higher risk of gout than the homozygous major genotypes with pooled OR_1_ and OR_2_ of 4.53 (4.10, 5.00) and 2.10 (1.95, 2.26) for rs2231142; and 3.86 (2.30, 9.76) and 2.46 (1.93, 3.18) for rs72552713. Sensitivity analysis by including a study that did not comply with HWE for rs2231142 [42] did not change the results (data not shown). Likewise, rs2231142 also carried higher risk of gout in Caucasians with pooled OR_1_ and OR_2_ of 3.24 (2.39, 4.41) and 1.64 (1.47, 1.82). Conversely, carrying homozygous minor and heterozygous genotypes of rs2231137 carried lower risk of gout in Asians with pooled OR_1_ and OR_2_ of 0.43 (0.34, 0.55) and 0.64 (0.56, 0.72), respectively.

**Table 1:** Summary of pooled effect sizes of *ABCG2* and *SLC2A9* polymorphisms on gout and hyperuricemia

	Asian						Caucasian					
	N^a^	I^2^	Pooled OR	95% CI	Lambda^b^	95% CI	N^a^	I^2^	Pooled OR	95% CI	Lambda^b^	95% CI
***ABCG2***												
**Gout**												
rs2231142												
OR_1_ (AA versus CC)	16	75.2	4.53	4.10, 5.00	0.463	0.332, 0.616	5	39.2	3.24	2.39, 4.41	0.461	0.253, 0.883
OR_2_ (CA versus CC)	16	68.7	2.10	1.95, 2.26			5	62.9	1.64	1.47, 1.82		
rs72552713												
OR_1_ (TT versus CC)	8	0.0	3.86	2.30, 9.76	0.670	0.362, 0.978						
OR_2_ (CT versus CC)	8	0.0	2.46	1.93, 3.18								
rs2231137												
OR_1_ (AA versus GG)	6	54.6	0.43	0.34, 0.55	0.547	0.262, 0.925						
OR_2_ (GA versus GG)	6	54.9	0.64	0.56, 0.72								
**Hyperuricemia**												
rs2231142												
OR_1_ (AA versus CC)	3	67.2	2.25	1.80, 2.81	0.527	0.131, 0.940						
OR_2_ (CA versus CC)	3	44.6	1.56	1.35, 1.80								
***SLC2A9***												
**Gout**												
rs1014290												
OR_1_ (CC versus TT)	6	55.0	0.42	0.36, 0.49	0.520	0.317, 0.847	3	0.0	0.57	0.37, 0.87	0.474	0.092, 0.954
OR_2_ (TC versus TT)	6	0.0	0.65	0.58, 0.72			3	0.0	0.75	0.63, 0.89		
rs3733591												
OR_1_ (CC versus TT)	6	68.1	1.55	1.17, 2.06	0.512	0.087, 0.960	3	0.0	1.03	0.60, 1.79	0.463	0.021, 0.972
OR_2_ (TC versus TT)	6	45.2	1.29	1.07, 1.55			3	28.8	0.85	0.48, 1.50		
rs6449213												
OR_1_ (CC versus TT)							5	29.0	0.38	0.29, 0.50	0.400	0.179, 0.829
OR_2_ (TC versus TT)							5	0.0	0.69	0.62, 0.78		
rs6855911												
OR_1_ (GG versus AA)	3	0.0	0.61	0.20, 1.87	0.395	0.019, 0.963	2	40.0	0.36	0.28, 0.46	0.370	0.078, 0.872
OR_2_ (AG versus AA)	3	0.0	0.82	0.53, 1.27			2	49.4	0.67	0.59, 0.77		
rs16890979												
OR_1_ (TT versus CC)	3	NA^c^	NA^c^									
OR_2_ (CT versus CC)	3	0.0	0.60	0.39, 0.94								
rs7442295												
OR_1_ (GG versus AA)							3	38.7	0.35	0.28, 0.46	0.401	0.163, 0.799
OR_2_ (AG versus AA)							3	8.6	0.65	0.57, 0.74		
rs12510549												
OR_1_ (CC versus TT)							3	0.0	0.44	0.34, 0.58	0.528	0.216, 0.942
OR_2_ (TC versus TT)							3	8.5	0.66	0.58, 0.76		

*ABCG2* ATP-binding cassette sub-family G member 2, *CI* confidence interval, *NA* not applicable, *OR* odds ratio, *SLC2A9* solute carrier family 2 member 9

^**a**^Number of sub-studies

^b^Median lambda

^c^Unable to pool OR_1_ for rs16890979 due to no gout case with TT genotype found

Effects of 7 polymorphisms in *SLC2A9* (i.e., rs1014290 (*N* = 7), rs3733591 (*N* = 7), rs6449213 (*N* = 5), rs6855911 (*N* = 5), rs16890979 (*N* = 4), rs7442295 (*N* = 3), and rs12510549 (*N* = 3)) on gout were assessed, see Fig. 2, Table 1, and Additional file 4.1. Pooled minor allele prevalences are reported in Additional file 4.2. Among Asian studies, effects of 3 polymorphisms (i.e., rs3733591, rs1014290, and rs6855911) on gout were pooled indicating that homozygous minor and heterozygous genotypes of rs3733591 carried higher risks of gout, i.e. 1.55 (1.17, 2.06) and 1.29 (1.07, 1.55) times higher than homozygous major genotype. Conversely, carrying homozygous minor and heterozygous genotypes of rs1014290 and rs6855911 carried lower risk, but only the former SNP was significant with pooled OR_1_ and OR_2_ of 0.42 (0.36, 0.49) and 0.65 (0.58, 0.72), respectively.

Among these *SLC2A9* polymorphisms, only rs1014290 and rs6855911 were significantly associated with gout in Caucasians with pooled OR_1_ and OR_2_ for homozygous minor and heterozygous genotypes of 0.57 (0.37, 0.87) and 0.75 (0.63, 0.89) for rs1014290, and 0.36 (0.28, 0.46) and 0.67 (0.59, 0.77) for rs6855911; the effects of rs3733591 were not significant. Three additional polymorphisms, studied only in Caucasians, were also significantly associated with gout, i.e., rs6449213, rs7442295, and rs12510549. Homozygous minor and heterozygous genotypes of these polymorphisms carried lower risk than homozygous major genotype, with pooled OR_1_ and OR_2_ of 0.38 (0.29, 0.50) and 0.69 (0.62, 0.78) for rs6449213; 0.35 (0.28, 0.46) and 0.65 (0.57, 0.74) for rs7442295; and 0.44 (0.34, 0.58) and 0.66 (0.58, 0.76) for rs12510549, see Fig. 2, Table 1, and Additional file 4.1.

The mode of gene effects (λ) were estimated suggesting that effects of *ABCG2* polymorphisms were mostly additive effects, except for rs72552713 which might be between an additive or dominant effect, see Table 1. Likewise, the mode of *SLC2A*9 effects on gout might be mostly additive effects, see Table 1.

Effects of *ABCG2* and *SLC2A9* polymorphisms on gout were homogenous to highly heterogeneous with I^2^ values of 0 to 75.2% and 39.2 to 62.9% in Asians and Caucasians for *ABCG2*; and 0 to 68.1% and 0 to 49.4% in Asians and Caucasians for *SLC2A9* polymorphisms, see Table 1. Sources of heterogeneity were explored for *ABCG2* polymorphisms (i.e., rs2231142 and rs2231137), see Additional file 4.3. Sub-group analysis by percent male ≥90% versus < 90% indicated stronger effects particularly for OR_1_ in percent male ≥90% with the OR_1_ of 5.32 (4.75, 5.97) and 6.14 (0.72, 52.13) for rs2231142 in Asians and Caucasians.

Likewise, sources of heterogeneity were explored for *SLC2A9* polymorphisms; again, percent male was a potential source of variation for rs1014290 and rs3733591 in Asians, and rs7442295 in Caucasians, see Additional file 4.3. Their effects were even higher in studies with a high percent of males, with pooled OR_1_ of 0.39 (0.33, 0.46) for rs1014290 and 2.52 (1.71, 3.73) for rs3733591 in Asians, and 0.19 (0.09, 0.41) for rs7442295 in Caucasians. Publication bias was assessed by Egger’s tests and funnel plots, see Additional file 4.4–4.5, and none of these polymorphisms had evidence of publication bias.

### Hyperuricemia

**Fig. 3:**
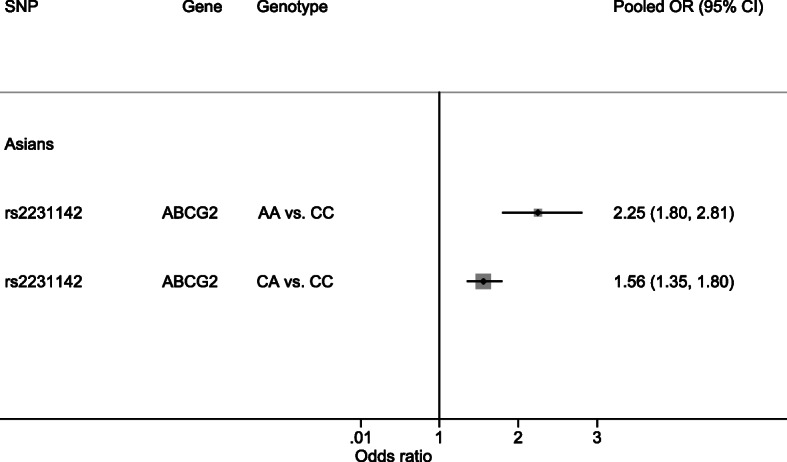
Summary of pooled genotype risk effects on hyperuricemia. *ABCG2*, ATP-binding cassette sub-family G member 2; CI, confidence interval; OR, odds ratio; SNP, single nucleotide polymorphism

Effects of rs2231142 (in *ABCG2*) on hyperuricemia were assessed (*N* = 5, see Additional file 5.1). Carrying homozygous minor and heterozygous genotypes carried a higher risk of hyperuricemia than carrying homozygous major genotype with pooled OR_1_ and OR_2_ of 2.25 (1.80, 2.81) and 1.56 (1.35, 1.80), respectively, see Fig. 3, Table 1, and Additional file 5.1. The estimated λ was 0.527, suggesting an additive gene effect, see Table 1. Sensitivity analysis by including 1 Asian study that did not comply with HWE [42] did not materially change the results (data not shown). These gene effects were moderately heterogeneous (I^2^ = 44.6–67.2%), with BMI and percent male as potential sources, see Additional file 5.2. There was no evidence of publication bias from Egger’s tests and funnel plots, see Additional file 5.3–5.4.

### Serum urate

**Fig. 4:**
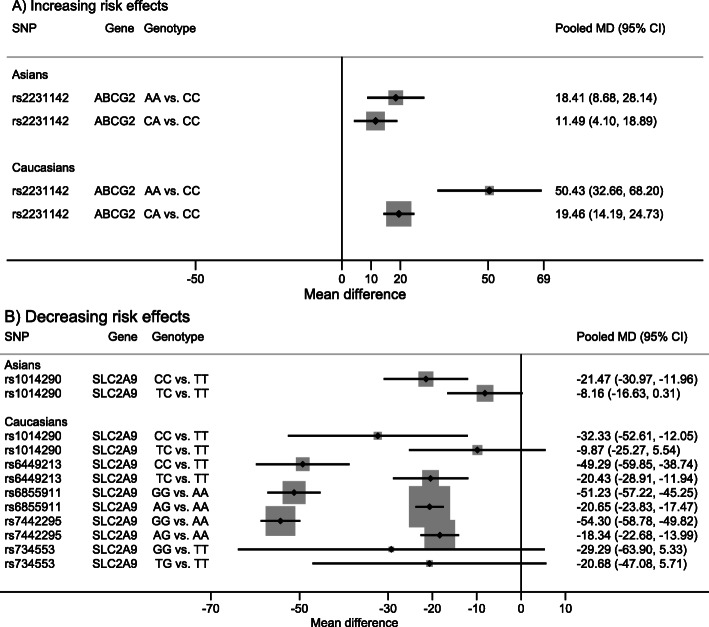
Summary of pooled genotype effects on mean difference of serum urate. **a** Increasing risk effects **b** Decreasing risk effects. *ABCG2*, ATP-binding cassette sub-family G member 2; CI, confidence interval; MD, mean difference; *SLC2A9*, solute carrier family 2 member 9; SNP, single nucleotide polymorphism

Among 22 studies, 1 SNP in *ABCG2* (i.e., rs2231142) and 5 SNPs in *SLC2A9* (i.e., rs1014290, rs6449213, rs6855911, rs7449253, and rs734553) had sufficient data for pooling, see Additional file 6.1. An additional polymorphism not studied for gout was rs734553 and the pooled minor allele prevalence was 0.30 (0.28, 0.32). For rs2231142 (in *ABCG2*), homozygous minor and heterozygous genotypes had higher urate levels than homozygous major genotype, with pooled MD_1_ and MD_2_ of 18.41 (8.68, 28.14) and 11.49 (4.10, 18.89) respectively in Asians; and 50.43 (32.66, 68.20) and 19.46 (14.19, 24.73) respectively in Caucasians, see Fig. 4, Table 2, and Additional file 6.1. Sensitivity analysis by including 1 Asian study that did not comply with HWE [39] did not materially change the results (data not shown). In contrast, homozygous minor and heterozygous genotypes of rs1014290 (in *SLC2A9*) decreased urate more than homozygous major genotype, with pooled MD_1_ and MD_2_ of − 21.47 (− 30.97, − 11.96) and − 8.16 (− 16.63, 0.31), respectively in Asians, and − 32.33 (− 52.61,-12.05) and − 9.87 (− 25.27,5.54), respectively in Caucasians, see Fig. 4, Table 2, and Additional file 6.1.

**Table 2:** Summary of pooled effect sizes of *ABCG2* and *SLC2A9* polymorphisms on serum urate

	Asian						Caucasian					
	N^a^	I^2^	Pooled MD	95% CI	Lambda^b^	95% CI	N^a^	I^2^	Pooled MD	95%CI	Lambda^b^	95% CI
***ABCG2***												
**Serum urate**												
rs2231142												
MD_1_ (AA versus CC)	5	63.1	18.41	8.68, 28.14	0.605	0.284, 0.956	4	68.7	50.43	32.66, 68.20	0.411	0.197, 0.783
MD_2_ (CA versus CC)	5	59.1	11.49	4.10, 18.89			4	0.0	19.46	14.19, 24.73		
***SLC2A9***												
**Serum urate**												
rs1014290												
MD_1_ (CC versus TT)	6	67.9	−21.47	−30.97, −11.96	0.381	0.088, 0.856	3	86.9	−32.33	−52.61,-12.05	0.420	0.087, 0.932
MD_2_ (TC versus TT)	6	65.7	−8.16	−16.63, 0.31			3	75.1	−9.87	−25.27,5.54		
rs6449213												
MD_1_ (CC versus TT)							6	88.5	−49.29	−59.85, −38.74	0.375	0.239, 0.622
MD_2_ (TC versus TT)							6	26.0	−20.43	−28.91, −11.94		
rs6855911												
MD_1_ (GG versus AA)							6	0.0	−51.23	−57.22, −45.25	0.399	0.316, 0.488
MD_2_ (AG versus AA)							6	0.0	−20.65	−23.83, −17.47		
rs7442295												
MD_1_ (GG versus AA)							6	0.0	−54.30	−58.78, − 49.82	0.326	0.280, 0.426
MD_2_ (AG versus AA)							6	26.4	−18.34	−22.68, −13.99		
rs734553												
MD_1_ (GG versus TT)							3	80.6	−29.29	−63.90, 5.33	0.592	0.152, 0.969
MD_2_ (TG versus TT)							3	52.8	−20.68	−47.08, 5.71		

*ABCG2* ATP-binding cassette sub-family G member 2, *CI* confidence interval, *MD* mean difference, *SLC2A9* solute carrier family 2 member 9

^a^Number of sub-studies

^b^Median lambda

Four additional *SLC2A9* polymorphisms were studied in Caucasians only. Homozygous minor and heterozygous genotypes of these SNPs reduced urate levels compared to homozygous major genotypes, with pooled MD_1_ and MD_2_ of − 49.29 (− 59.85, − 38.74) and − 20.43 (− 28.91, − 11.94) for rs6449213; − 51.23 (− 57.22, − 45.25) and − 20.65 (− 23.83, − 17.47) for rs6855911; − 54.30 (− 58.78, − 49.82) and − 54.30 (− 58.78, − 49.82) for rs7442295; − 29.29 (− 63.90, 5.33) and − 20.68 (− 47.08, 5.71) for rs734553, see Fig. 4, Table 2, and Additional file 6.1. The mode of effects of these *SLC2A9*-SNPs were most likely to be an additive effect, see Table 2.

Effects of *ABCG2* and *SLC2A9* SNPs on serum urate were homogeneous to highly heterogeneous, i.e., I^2^ ranged from 59.1 to 63.1% and 0 to 68.7% in Asians and Caucasians for *ABCG2*-rs2231142; 65.7 to 67.9% and 0 to 88.5% in Asians and Caucasians for *SLC2A9* SNPs, see Table 2. In Caucasians, type of population accounted for some of the heterogeneity, see Additional file 6.2. After excluding one Caucasian study in type 2 diabetes (T2D) patients, sub-group analysis yielded stronger effects of these SNPs in a general population with pooled MD_1_ and MD_2_ of − 40.16 (− 47.74, − 32.57) and − 15.80 (− 23.36, − 8.24) for rs1014290; and − 55.67 (− 82.77, − 28.57)) and − 29.00 (− 41.82, − 16.19) for rs734553. The effects of rs734553 became significant and strongest after excluding the study in the T2D population. Publication bias was assessed by Egger’s tests and funnel plots, see Additional file 6.3–6.4. Publication bias may be present for the effects of rs6449213 in Caucasians, as suggested by asymmetry of the funnel and the contour enhanced-funnel plot (data not shown). The pooled effect sizes of *ABCG2* and *SLC2A9* SNPs on all outcomes are summarized in Table 3.

**Table 3:** Summary of pooled effect sizes of *ABCG2* and *SLC2A9* polymorphisms on all outcomes^a^

	Ethnicity	Gout				Hyperuricemia				Serum urate			
		Pooled OR_1_	95% CI	Pooled OR_2_	95% CI	Pooled OR_1_	95% CI	Pooled OR_2_	95% CI	Pooled MD_1_	95% CI	Pooled MD_2_	95% CI
***ABCG2***													
rs2231142	Asian	**4.53**	**4.10, 5.00**	**2.10**	**1.95, 2.26**	**2.25**	**1.80, 2.81**	**1.56**	**1.35, 1.80**	**18.41**	**8.68, 28.14**	**11.49**	**4.10, 18.89**
	Caucasian	**3.24**	**2.39, 4.41**	**1.64**	**1.47, 1.82**					**50.43**	**32.66, 68.20**	**19.46**	**14.19, 24.73**
rs72552713	Asian	**3.86**	**2.30, 9.76**	**2.46**	**1.93, 3.18**								
rs2231137	Asian	**0.43**	**0.34, 0.55**	**0.64**	**0.56, 0.72**								
***SLC2A9***													
rs1014290	Asian	**0.42**	**0.36, 0.49**	**0.65**	**0.58, 0.72**					**−21.47**	**−30.97, −11.96**	−8.16	−16.63, 0.31
	Caucasian	**0.57**	**0.37, 0.87**	**0.75**	**0.63, 0.89**					**−32.33**	**−52.61,-12.05**	**−9.87**	**−25.27,5.54**
rs3733591	Asian	**1.55**	**1.17, 2.06**	**1.29**	**1.07, 1.55**								
	Caucasian	1.03	0.60, 1.79	0.85	0.48, 1.50								
rs6855911	Asian	0.61	0.20, 1.87	0.82	0.53, 1.27								
	Caucasian	**0.36**	**0.28, 0.46**	**0.67**	**0.59, 0.77**					**−51.23**	**−57.22, −45.25**	**−20.65**	**−23.83, −17.47**
rs6442295	Caucasian	**0.38**	**0.29, 0.50**	**0.69**	**0.62, 0.78**					**−49.29**	**−59.85, −38.74**	**−20.43**	**−28.91, −11.94**
rs7442295	Caucasian	**0.35**	**0.28, 0.46**	**0.65**	**0.57, 0.74**					**−54.30**	**−58.78, −49.82**	**−18.34**	**−22.68, −13.99**
rs12510549	Caucasian	**0.44**	**0.34, 0.58**	**0.66**	**0.58, 0.76**								
rs16890979	Asian	NA^b^		**0.60**	**0.39, 0.94**								
rs734553	Caucasian									−29.29	−63.90, 5.33	−20.68	−47.08, 5.71

*ABCG2* ATP-binding cassette sub-family G member 2, *CI* confidence interval, *MD* mean difference, *NA* not applicable, *OR* odds ratio, *SLC2A9* solute carrier family 2 member 9

^a^Significant OR or MD in bold

^b^Unable to pool OR_1_ for rs16890979 due to no gout case with homozygous minor genotype found

## Discussion

We conducted a systematic review and meta-analysis to assess associations between SNPs in *ABCG2* and *SLC2A9* and gout, hyperuricemia and serum urate, stratified by ethnicity. *ABCG2*-SNPs were common in both Asians and Caucasians with a minor allele frequency of 11 to 31%, except for rs72552713 which was very rare in Asians*. SLC2A9*-SNPs were common in both Asians (6–41%) and Caucasians (18–40%), except for rs6855911 and rs16890979 which were rare in Asians (1–6%). For *ABCG2*, rs2231142 significantly increased risk of gout by about 2 to 4 times and 1.6 to 3 times in Asians and Caucasians respectively for carrying heterozygous and homozygous minor genotypes. In addition, carrying homozygous/heterozygous minor genotypes of rs72552713 also increased risk of gout by about 2.5–3.9 times in Asians. By contrast, carrying homozygous minor or heterozygous genotypes of rs2231137 reduced risk of gout by about 36–57% in Asians. Likewise, most *SLC2A9*-SNPs (i.e., rs1014290, rs6449213, rs6855911, rs7442295, and rs12510549) showed significantly lower risk of gout by about 25–65% in Asians and/or Caucasians, except for rs6855911 which was not significant in Asians. Carrying homozygous minor or heterozygous genotypes of rs2231142-*ABCG2* also increased the risk of hyperuricemia in Asians and serum urate in both Asians and Caucasians. In addition, all *SLC2A9*-SNPs, except rs1014290 and rs734553, also significantly increased serum urate level.

Regarding *ABCG2*, risk effects of rs2231142 were consistently found on gout, hyperuricemia and increased serum urate in Asians. The risk effects were strong on gout compared to weaker effects on hyperuricemia and modest effects on urate level. There might be other mechanisms of rs2231142 that lead to gout occurrence without raising serum urate level. In Caucasians, there were weaker risk effects of rs2231142 on gout, but stronger risk effects on serum urate.

For *SLC2A9*, rs6855911, rs6449213 and rs7442295 could significantly lower risk of gout (OR_1_ of 0.35–0.38 and OR_2_ of 0.65–0.69) and serum urate (MD_1_ of − 54.30 to − 49.29 and MD_2_ of − 20 .65 to − 18.34) in Caucasians. The causative SNP might be any of these 3 SNPs because there was high linkage disequilibrium between rs6449213 and rs7442295 (r^2^ = 0.88) [31]. rs1014290 also lowered the risk of gout in Caucasians and Asians, and its effects on serum urate were significant in Asians and for MD_1_ in Caucasians; the lack of significance in MD_2_ in Caucasians is likely due to the low numbers of included studies in the latter given similar risk effects.

The percentage of males might be a potential source of heterogeneity in the effects of *ABCG2* and *SLC2A9* on gout, since the risk or protective effects were consistently stronger in men; this may indicate sex-specific differences in pathological mechanisms or in the handling of urate. T2D might be another source of heterogeneity (for the outcome of serum urate); excluding the study with T2D Caucasians [26], made the effects of rs1014290 and rs734553 stronger in the remaining/general population.

Considering the effect of *ABCG2* SNPs on gout, effects of rs2231142 were consistent with the recent updated meta-analyses that pooled allele [12] and genotype [11] effects. Our pooling had 12 additional studies and stratified by Asian and Caucasian ethnicity because of different allele frequencies across these populations. Our results for *SLC2A9* SNPs and gout were also generally consistent with the recent updated meta-analysis, but we were able to pool additional effects of rs1014290, rs6449213 and rs7449213 in Caucasians and also rs6855911 in Asians. Some previous reviews reported only pooled allele effects [12, 15] and some reported many different types of pooling effects [14, 16]. However, we are the first to report both pooled homozygous and heterozygous effects of these *SLC2A9* SNPs on gout. Furthermore, we also explored the effects of rs72552713 on gout, rs2231142 on hyperuricemia, and SNPs of both genes on serum urate level. Risk of bias in ascertainment of genotyping and outcome were observed. However, publication bias was not evidenced in most analyses.

The ABCG2 protein is a urate efflux transporter responsible for urate excretion [42, 46]. In vitro studies showed that rs2231142 reduced ABCG2 protein expression [42, 73–75], ATPase activity [76] and the urate transport [21, 42, 46] resulting in higher accumulation of urate whereas rs2231137 did not change the level of ABCG2 protein expression [42, 73, 75] and the urate transport activity [21, 42]. The proposed mechanism by which rs2231137 lowers the risk of gout is still unknown. The effect of rs72552713 on increasing the risk of gout is supported by the fact that it is a nonsense variant causing a stop codon that prematurely ends protein translation of ABCG2; Matsuo et al. [42] also reported the absence of ABCG2 protein expression and the almost entirely removed urate transport activity due to rs72552713 in the functional analysis. It was also found that the combination of rs2231142 and rs72552713 attributed to a higher risk of hyperuricemia than other typical risk factors including obesity, alcohol consumption and increasing age [77]. *ABCG2* variants were associated with gout/hyperuricemia in both Asians and Caucasians [21, 78]. Particularly, individuals of European descent who carried *ABCG2* variants had a significantly earlier onset of gout/hyperuricemia and the family history of gout was significantly more frequent among them [78]. These findings are supported by a recent study showing that pediatric-onset gout/hyperuricemia patients had higher minor allele frequency of rs2231142 than those of adult-onset patients and normouricemic controls [79].

The glucose transporter 9 (GLUT9) protein, regulated by the *SLC2A9* gene, is a urate reuptake transporter [3]. It was discovered that *SLC2A9* polymorphisms cause renal hypouricemia type 2 by reducing the urate transport activity leading to lowering renal urate reabsorption and lower serum urate [80–82]. Therefore, *SLC2A9* could be another promising target for lowering serum urate and the risk of gout. However, one study found that *SLC2A9* variants including rs16890979 and rs3733591 did not significantly change the urate transport activity in *Xenopus* oocyte model [83]. Therefore, the findings regarding the functional mechanisms underlying the GWAS-identified association of *SLC2A9* variants on urate transport are still not consistent. Our pooled results showed a trend of decreasing the risk of gout and serum urate in most *SLC2A9* variants, except for the effect of rs3733591 on gout in Asians. More association and functional studies are required to confirm/explain this. In addition, a recent GWAS found that *ABCG2, SLC2A9, ALDH2,* and novel loci induce asymptomatic hyperuricemia into gout development [84]. This emphasizes the strong influence of *ABCG2* and *SLC2A9* variants on gout development and points out the importance of identifying high risk patients having these genetic variants.

Our study has some strengths. We pooled genotype effects stratified by ethnicity for different allele frequencies. We considered all relevant outcomes, i.e., gout, hyperuricemia, and serum urate and mode of gene effects were estimated. Although some gene effects were heterogeneous, we could identify that male gender and diabetes might be potential sources of heterogeneity. However, we could not avoid some limitations. Genotype frequencies in some studies [23, 24, 26, 27, 32, 34, 38, 48, 53, 56, 61, 67, 68, 70] were missing and thus were estimated assuming HWE. Some of these SNPs were in high linkage disequilibrium, so haplotype effects should be further assessed to determine which polymorphism had the largest causal effect. Other important confounders (e.g. diuretic use, dietary intake and alcohol consumption) of gout/urate should be considered in assessing any association, but these data were not available. Only a few number of studies conducted in African Americans and other indigenous people, so further studies in these populations should be conducted.

In conclusion, our study suggested that the *ABCG2*-rs2231142 SNP increased the risk of gout and serum urate in both Asians and Caucasians. In addition, *ABCG2*-rs72552713 also increased the risk of gout whereas *ABCG2*-rs2231137 reduced the risk in Asians. Furthermore, *SLC2A9*-SNPs (i.e., rs1014290, rs6449213, rs6855911, rs7442295, and rs12510549) significantly lowered the risk of gout in Asians and/or Caucasians. Further studies in other populations especially African Americans are still needed. Our findings may be helpful in creating more accurate risk stratification models.

## Supplementary information

Additional file 1. Search strategies. (DOCX 26 kb)

Additional file 2. Characteristics of included studies investigating associations between *ABCG2* and *SLC2A9* polymorphisms and urate. (DOCX 146 kb)

Additional file 3. Risk of bias assessment. (DOCX 26 kb)

Additional file 4. Gout. **4.1**. Data used for pooling effects of *ABCG2* and *SLC2A9* polymorphisms on gout. **4.2**. Pooled prevalence of minor allele of *ABCG2* and *SLC2A9* polymorphisms. **4.3**. Exploring source of heterogeneity for *ABCG2* and *SLC2A9* polymorphisms on gout.**4.4**. Egger’s tests for *ABCG2* and *SLC2A9* polymorphisms on gout. **4.5**. Funnel plots for *ABCG2* and *SLC2A9* polymorphisms on gout. (DOCX 662 kb)

Additional file 5. Hyperuricemia. **5.1**. Data used for pooling effects of *ABCG2*-rs2231142 on hyperuricemia. **5.2**. Exploring source of heterogeneity for *ABCG2*-rs2231142 on hyperuricemia. **5.3**. Egger’s tests for *ABCG2*-rs2231142 on hyperuricemia. **5.4**. Funnel plots of *ABCG2*-rs2231142 on hyperuricemia in Asians. A) OR_1_ in Asians B) OR_2_ in Asians. (DOCX 77 kb)

Additional file 6. Serum urate. **6.1**. Data used for pooling mean difference of *ABCG2* and *SLC2A9* polymorphisms on serum urate. **6.2**. Exploring source of heterogeneity for *ABCG2* and *SLC2A9* polymorphisms on serum urate. **6.3**. Egger’s tests for *ABCG2* and *SLC2A9* polymorphisms on serum urate. **6.4**. Funnel plots for *ABCG2* and *SLC2A9* polymorphisms on serum urate. (DOCX 412 kb)

## Data Availability

All data generated or analysed during this study are included in this published article and its supplementary information files.

## Abbreviations

*ABCG2*: ATP-binding cassette sub-family G member 2
BMI: Body mass index
CI: Confidence interval
eGFR: Estimated glomerular filtration rate
GWAS: Genome-wide association study
HWE: Hardy-Weinberg equilibrium
MD: Mean difference
OR: Odds ratio
SD: Standard deviation
*SLC2A9*: Solute carrier family 2 member 9
SNP: Single nucleotide polymorphism
T2D: Type 2 diabetes
