# Supplementary material for: The association between genetic polymorphisms in ABCG2 and SLC2A9 and urate: an updated systematic review and meta-analysis
Source: BMC Med Genet. 2020 Oct 21;21:210. doi: 10.1186/s12881-020-01147-2 (PMC7580000; doi:10.1186/s12881-020-01147-2)
Supplement: Supplementary file 1 — Search strategies. (DOCX 26 kb) [file 12881_2020_1147_MOESM1_ESM.docx]

# Supplementary information files

Additional file 1. Search strategies

Additional file 2. Characteristics of included studies investigating associations between *ABCG2* and *SLC2A9* polymorphisms and urate

Additional file 3. Risk of bias assessment

Additional file 4. Gout

Additional file 4.1. Data used for pooling effects of *ABCG2* and *SLC2A9* polymorphisms on gout

Additional file 4.2. Pooled prevalence of minor allele of *ABCG2* and *SLC2A9* polymorphisms

Additional file 4.3. Exploring source of heterogeneity for *ABCG2* and *SLC2A9* polymorphisms on gout

Additional file 4.4. Egger’s tests for *ABCG2* and *SLC2A9* polymorphisms on gout

Additional file 4.5. Funnel plots for *ABCG2* and *SLC2A9* polymorphisms on gout

Additional file 5. Hyperuricemia

Additional file 5.1. Data used for pooling effects of *ABCG2*-rs2231142 on hyperuricemia

Additional file 5.2. Exploring source of heterogeneity for *ABCG2*-rs2231142 on hyperuricemia

Additional file 5.3. Egger’s tests for *ABCG2*-rs2231142 on hyperuricemia

Additional file 5.4. Funnel plots of *ABCG2*-rs2231142 on hyperuricemia in Asians. A) OR_1_ in Asians B) OR_2_ in Asians

Additional file 6. Serum urate

Additional file 6.1. Data used for pooling mean difference of *ABCG2* and *SLC2A9* polymorphisms on serum urate

Additional file 6.2. Exploring source of heterogeneity for *ABCG2* and *SLC2A9* polymorphisms on serum urate

Additional file 6.3. Egger’s tests for *ABCG2* and *SLC2A9* polymorphisms on serum urate

Additional file 6.4. Funnel plots for *ABCG2* and *SLC2A9* polymorphisms on serum urate

# Additional file 1. Search strategies

1. **Search strategies in Medline**

| **Domain** | | **Search** | **Query** | **Items found** |
| --- | --- | --- | --- | --- |
| Interventions and outcomes | #44 | | #34 AND #43 | 271 |
| Outcomes | #43 | | #35 OR #36 OR #37 OR #38 OR #39 OR #42 | 51756 |
|  | #42 | | hypouricemia OR hypouricaemia | 566 |
|  | #41 | | hypouricaemia | 71 |
|  | #40 | | hypouricemia | 503 |
|  | #39 | | *uric* | 37173 |
|  | #38 | | hyperuricemia | 7706 |
|  | #37 | | urate | 37833 |
|  | #36 | | "gouty arthritis" | 1928 |
|  | #35 | | gouts | 16712 |
| Interventions | #34 | | #32 AND #33 | 2837 |
|  | #33 | | #10 OR #17 | 5893 |
| Intervention (gene domain) | #32 | | #18 OR #19 OR #20 OR #21 OR #22 OR #23 OR #24 OR #25 OR #26 OR #27 OR #28 OR #29 OR #30 OR #31 | 2710587 |
|  | #31 | | "nucleotide polymorphisms, single" | 122008 |
|  | #30 | | snps | 123405 |
|  | #29 | | snp | 50489 |
|  | #28 | | "gene variations" | 775 |
|  | #27 | | "gene variation" | 1149 |
|  | #26 | | "gene variants" | 6024 |
|  | #25 | | "gene variant" | 1780 |
|  | #24 | | "genetic variants" | 21328 |
|  | #23 | | "genetic variant" | 3578 |
|  | #22 | | "genetic variations" | 8008 |
|  | #21 | | "genetic variation" | 124856 |
|  | #20 | | genotypes | 465990 |
|  | #19 | | allele | 238260 |
|  | #18 | | gene | 2464690 |
| Intervention 2 (*SLC2A9* gene) | #17 | | #11 OR #12 OR #13 OR #14 OR #15 OR #16 | 809 |
|  | #16 | | "glucose transporter 9" | 62 |
|  | #15 | | glut-9 | 10 |
|  | #14 | | glut9 | 145 |
|  | #13 | | slc2a9* | 273 |
|  | #12 | | "solute carrier family 2 member 9" | 485 |
|  | #11 | | slc2a9 | 270 |

1. **Search strategies in Medline (cont.)**

| **Domain** | **Search** | **Query** | **Items found** |
| --- | --- | --- | --- |
| Intervention 1 (*ABCG2* gene) | #10 | #1 OR #2 OR #3 OR #4 OR #5 OR #6 OR #7 OR #8 OR #9 | 5203 |
|  | #9 | rs2231142 | 86 |
|  | #8 | Q141K | 75 |
|  | #7 | C421A | 51 |
|  | #6 | bcrp/abcg2 | 308 |
|  | #5 | abcg2/bcrp | 87 |
|  | #4 | "breast cancer resistance protein" | 1742 |
|  | #3 | bcrp | 2280 |
|  | #2 | abcg2 | 4203 |
|  | #1 | "ATP Binding Cassette Transporter, Sub-Family G, Member 2" | 2651 |

1. **Search strategies in Scopus**

| **Domain** | **Search** | **Query** | **Items found** |
| --- | --- | --- | --- |
| Interventions AND outcomes | #40 | #30 AND #39 | 1816 |
| Outcomes | #39 | #31 OR #32 OR #33 OR #34 OR #35 OR #36 OR #37 OR #38 | 23263 |
|  | #38 | hypouricaemia | 1744627 |
|  | #37 | hypouricemia | 2139 |
|  | #36 | hyperuricaemia | 2009 |
|  | #35 | hyperuricemia | 25983 |
|  | #34 | "uric acid" | 24192 |
|  | #33 | urate | 114196 |
|  | #32 | "gouty arthritis" | 30857 |
|  | #31 | gout | 75888 |
| Interventions | #30 | #28 AND #29 | 75887 |
|  | #29 | #8 OR #16 | 75888 |

1. **Search strategies in Scopus (cont.)**

| **Domain** | **Search** | **Query** | **Items found** |
| --- | --- | --- | --- |
| Intervention (gene domain) | #28 | #17 OR #18 OR #19 OR #20 OR #21 OR #22 OR #23 OR #24 OR #25 OR #26 OR #27 | 6512548 |
|  | #27 | snps | 102808 |
|  | #26 | snp | 171483 |
|  | #25 | "single nucleotide polymorphisms" | 233827 |
|  | #24 | "polymorphism, single nucleotide" | 94257 |
|  | #23 | "gene variant" | 55855 |
|  | #22 | "genetic variant" | 111526 |
|  | #21 | "gene variation" | 18174 |
|  | #20 | "genetic variation" | 347804 |
|  | #19 | genotype | 973844 |
|  | #18 | allele | 609020 |
|  | #17 | genes | 6092366 |
| Intervention 2 (*SLC2A9* gene) | #16 | #9 OR #10 OR #11 OR #12 OR #13 OR #14 OR #15 | 1927 |
|  | #15 | "glucose transporter 9" | 476 |
|  | #14 | glut-9 | 52 |
|  | #13 | glut9 | 1038 |
|  | #12 | "solute carrier family 2 member 9" | 40 |
|  | #11 | slc2a9b | 34 |
|  | #10 | slc2a9a | 32 |
|  | #9 | slc2a9 | 1285 |
| Intervention 1 (*ABCG2* gene) | #8 | #1 OR #2 OR #3 OR #4 OR #5 OR #6 OR #7 | 27262 |
|  | #7 | rs2231142 | 142 |
|  | #6 | q141k | 559 |
|  | #5 | c421a | 525 |
|  | #4 | "breast cancer resistance protein" | 13826 |
|  | #3 | bcrp | 12355 |
|  | #2 | abcg2 | 21144 |
|  | #1 | "ATP Binding Cassette Transporter Sub-Family G Member 2" | 413 |
