# Supplementary material for: The association between genetic polymorphisms in ABCG2 and SLC2A9 and urate: an updated systematic review and meta-analysis
Source: BMC Med Genet. 2020 Oct 21;21:210. doi: 10.1186/s12881-020-01147-2 (PMC7580000; doi:10.1186/s12881-020-01147-2)
Supplement: Supplementary file 2 — Characteristics of included studies investigating associations between ABCG2 and SLC2A9 polymorphisms and urate. (DOCX 146 kb) [file 12881_2020_1147_MOESM2_ESM.docx]

# Additional file 2. Characteristics of included studies investigating associations between *ABCG2* and *SLC2A9* polymorphisms and urate

| **First Author, Year** | **Country** | **Study design** | **Types of subjects** | **Ethnicity** | **Gene** | **SNP** | **Outcome** | **Mean age** | **%Male** | **Mean BMI (kg/m^2^)** |
| --- | --- | --- | --- | --- | --- | --- | --- | --- | --- | --- |
| Li, 2007 [69] | USA |  |  |  |  |  |  |  |  |  |
| - InCHIANTI |  | Cohort | General | Caucasian | *SLC2A9* | rs6855911, rs7442295 | Urate |  | 45 |  |
| - SadiNIA |  | Cohort | General | Caucasian | *SLC2A9* | rs6855911, rs7442295 | Urate |  | 47 |  |
| Brandstatter, 2008 [65] | Austria |  |  |  |  |  |  |  |  |  |
| - Utah |  | Cohort | General | Caucasian | *SLC2A9* | rs6449213, rs6855911, rs7442295 | Urate | 52.8 | 48.5 | 27.6 |
| Doring, 2008 [68] | Germany |  |  |  |  |  |  |  |  |  |
| - KORA |  | Cohort | General | Caucasian | *SLC2A9* | rs6449213, rs6855911, rs7442295, rs12510549 | Gout | 53 | 49.4 |  |
| - SHIP |  | Cohort | General | Caucasian | *SLC2A9* | rs7442295 | Gout | 49.8 | 49.8 |  |
| Stark, 2008 [66] |  | Case-control | General | Caucasian | *SLC2A9* | rs6449213, rs7442295, rs12510549 | Gout | 59.9 | 72 | 27.6 |
| Vitart, 2008 [67] | UK |  |  |  |  |  |  |  |  |  |
| - Croatia |  | Cohort | General | Caucasian | *SLC2A9* | rs1014290, rs6449213 | Urate |  | 42.6 |  |
| - Croatia |  | Cohort | General | Caucasian | *SLC2A9* | rs1014290, rs6449213 | Gout |  | 39 |  |
| - Germany |  | Cohort | General | Caucasian | *SLC2A9* | rs1014290, rs6449213 | Gout |  | 54.6 |  |
| - Scotland   (Orkney) |  | Cohort | General | Caucasian | *SLC2A9* | rs1014290, rs6449213 | Urate |  | 44.7 |  |
| - Scotland   (Go-DARTS) |  | Cohort | General | Caucasian | *SLC2A9* | rs1014290, rs6449213 | Gout |  |  |  |
| Hollis-Moffatt, 2009 [63] | New Zealand |  |  |  |  |  |  |  |  |  |
|  |  | Case-control | General | Caucasian | *SLC2A9* | rs12510549, rs16890979 | Gout |  |  |  |
|  |  | Case-control | General | Polynesian | *SLC2A9* | rs12510549, rs16890979 | Gout |  |  |  |
| Matsuo, 2009 [42] | Japan |  |  |  |  |  |  |  |  |  |
|  |  | Case-control | General | Asian | *ABCG2* | rs2231142 | HUA |  | 100 |  |
|  |  | Cross-sectional | General | Asian | *ABCG2* | rs2231142 | Urate | 60.9 | 33.2 | 24.1 |
|  |  | Case-control | General | Asian | *ABCG2* | rs2231142, rs2231137, rs72552713 | Gout |  | 100 |  |
| Stark, 2009 [45] | Germany | Cohort | General | Caucasian | *ABCG2, SLC2A9* | rs2231142, rs6855911 | Gout | 58.4 | 60.2 | 27.2 |
| Woodward, 2009 [46] | USA | Cohort | General | Caucasian | *ABCG2* | rs2231142 | Gout | 54.1 | 46 | 26.9 |
| Brandstatter, 2010 [43] | Austria |  |  |  |  |  |  |  |  |  |
| - Bruneck |  | Cohort | General | Caucasian | *ABCG2, SLC2A9* | rs2231142, rs6449213, rs6855911, rs7442295 | Urate | 62.7 | 49.8 | 25.6 |
| - SAPHIR |  | Cohort | General | Caucasian | *ABCG2, SLC2A9* | rs2231142, rs6449213, rs6855911, rs7442295 | Urate | 51.8 | 63 | 26.8 |
| Cummings, 2010 [61] | Australia | Cohort | General | Mixed | *SLC2A9* | rs6449213, rs6855911, rs7442295 | Urate | 50.3 | 45.5 | 25 |
| Tabara, 2010 [40] | Japan |  |  |  |  |  |  |  |  |  |
| - Ehime |  | Cohort | General | Asian | *ABCG2, SLC2A9* | rs2231142, rs1014290 | Urate | 62 | 43.2 | 23 |
| - Suita |  | Cohort | General | Asian | *ABCG2, SLC2A9* | rs2231142, rs1014290 | Urate | 66 | 45.6 | 23 |
| Tu, 2010 [64] | Taiwan |  |  |  |  |  |  |  |  |  |
|  |  | Case-control | General | Asian | *SLC2A9* | rs37335591 | Gout | 55 | 100 |  |
|  |  | Case-control | General | Solomon Islander | *SLC2A9* | rs1014290, rs37335591 | Gout | 49 | 70.9 |  |
| Urano, 2010 [62] | Japan |  |  |  |  |  |  |  |  |  |
|  |  | Case-control | General | Asian | *SLC2A9* | rs1014290, rs6449213, rs6855911, rs7442295 | Urate |  | 100 |  |
|  |  | Case-control | General | Asian | *SLC2A9* | rs1014290, rs16890979, rs37335591, rs6449213, rs6855911, rs7442295 | Gout |  | 100 |  |
| Wang, 2010 [44] | China | Case-control | General | Asian | *ABCG2* | rs2231142 | Gout | 48.6 | 100 | 26.1 |
| Yamagishi, 2010 [41] | Japan |  |  |  |  |  |  |  |  |  |
|  |  | Cohort | General | Asian | *ABCG2* | rs2231142 | Urate | 62.1 | 39.1 |  |
|  |  | Cohort | General | Asian | *ABCG2* | rs2231142 | Gout | 62.1 | 39.1 |  |
| Guan, 2011 [60] | China |  |  |  |  |  |  |  |  |  |
|  |  | Case-control | General | Asian | *SLC2A9* | rs6855911 | Urate | 55.3 | 100 | 25.4 |
|  |  | Case-control | General | Asian | *SLC2A9* | rs6855911 | Gout | 55.3 | 100 | 25.4 |
| Hollis-Moffatt, 2011 [58] | New Zealand |  |  |  |  |  |  |  |  |  |
| - ARIC |  | Cohort | General | Caucasian | *SLC2A9* | rs3733591 | Gout | 54 | 45.6 | 27 |
| - FHS |  | Cohort | General | Caucasian | *SLC2A9* | rs3733591 | Gout | 37 | 45.7 | 27 |
| - NZ |  | Case-control | General | Caucasian | *SLC2A9* | rs3733591 | Gout | 50.3 | 56.6 | 28.7 |
|  |  | Case-control | General | Polynesian | *SLC2A9* | rs3733591 | Gout | 40.6 | 56.7 | 34 |
| Liu, 2011 [59] | Taiwan |  |  |  |  |  |  |  |  |  |
|  |  | Cross-sectional | General | Asian | *SLC2A9* | rs1014290 | Urate | 48.3 | 48.3 | 24.1 |
|  |  | Cross-sectional | General | Asian | *SLC2A9* | rs1014290 | Gout | 48.3 | 48.3 | 24.1 |
| Hu, 2012 [39] | Hong Kong | Cohort | Risk of CHD patients | Asian | *ABCG2, SLC2A9* | rs2231142, rs1014290 | Urate |  | 47 |  |
| Lyngdoh, 2012 [57] | Switzerland | Cross-sectional | General | Caucasian | *SLC2A9* | rs6855911 | Urate | 53.1 | 47.4 | 25.8 |
| Takeuchi, 2013 [38] | Japan |  |  |  |  |  |  |  |  |  |
|  |  | Case-control | General | Asian | *ABCG2* | rs2231142 | HUA | 51.7 | 44.6 | 22.6 |
| Urano, 2013 [48] | Japan | Case-control | General | Asian | *ABCG2* | rs2231142, rs72552713 | Gout | 40.3 | 100 | 23.6 |
| Voruganti, 2013 [56] | USA | Cohort | General | Caucasian | *SLC2A9* | rs6449213 | Urate | 47.9 |  |  |
| Stiburkova, 2014 [36] | Czech Republic | Cohort | General | Caucasian | *ABCG2* | rs2231142 | Urate | 49.3 | 48 | 25.9 |
| Testa, 2014 [55] | Italy | Cohort | General | Caucasian | *SLC2A9* | rs734553 | Urate |  | 43 |  |
| Tu, 2014 [37] | Taiwan |  |  |  |  |  |  |  |  |  |
| - 1 |  | Case-control | General | Asian  (Taiwanese Han) | *ABCG2* | rs2231142, rs2231137, rs72552713 | Gout | 53.2 | 96.6 | 25.2 |
| - 2 |  | Case-control | General | Asian (Taiwanese Aborigine) | *ABCG2* | rs2231142, rs2231137, rs72552713 | Gout | 51.8 | 52.3 | 26.4 |
| Wang, 2014 [49] | China | Case-control | General | Asian | *ABCG2* | rs2231142 | Gout | 61.5 | 0 | 25.3 |
| Zhou, 2014 [35] | China | Case-control | General | Asian | *ABCG2* | rs2231142, rs2231137, rs72552713 | Gout | 57.1 | 100 |  |
| Kim, 2015 [31] | Korea | Case-control | General | Asian | *ABCG2, SLC2A9* | rs2231142, rs6449213, rs16890979 | Gout | 53.5 | 98.8 | 24.3 |
| Laston, 2015 [53] | USA | Cohort | Subjects who had probands with CKD | American Indian | *SLC2A9* | rs6449213, rs734553 | Urate | 37.1 | 51.8 |  |
| Mahfudzah, 2015 [71] | Malaysia | Case-control | General | Asian | *SLC2A9* | rs3733591 | Gout |  | 100 |  |
| Mallamaci, 2015 [54] | Italy | Cross-sectional | General | Caucasian | *SLC2A9* | rs734553 | Urate | 41.9 | 46.2 | 27.6 |
| Wan, 2015 [33] | China | Case-control | General | Asian | *ABCG2, SLC2A9* | rs2231142, rs3733591 | Gout | 51 | 95 |  |
| Zhang, 2015 [52] | China | Cohort | Gout patients | Asian | *SLC2A9* | rs1014290 | Urate | 57.1 | 57.9 |  |
| Bartakova, 2016 [26] | Czech Republic | Cohort | T2D | Caucasian | *ABCG2, SLC2A9* | rs2231142, rs1014290, rs734553 | Urate |  | 51.5 |  |
| Jiri, 2016 [32] | China | Case-control | General | Asian | *ABCG2* | rs2231142 | Gout | 47.2 | 71.1 |  |
| Kannangara, 2016 [29] | New Zealand | Case-control | General | Mixed | *ABCG2* | rs2231142 | HUA | 53.2 | 69.3 | 31.4 |
| Matsuo, 2016 [34] | Japan | Case-control | General | Asian | *ABCG2* | rs2231142, rs72552713 | Gout | 48.4 | 100 | 24.3 |
| Oetjens, 2016 [27] | USA | Cohort | General | Caucasian and African | *ABCG2* | rs2231142 | Gout | 55 | 51.9 | 28.3 |
| Phipps-Green, 2016 [47] | New Zealand |  |  |  |  |  |  |  |  |  |
|  |  | Case-control | General | Caucasian | *ABCG2* | rs2231142 | Gout | 59.6 | 68.3 | 28 |
|  |  | Case-control | General | Polynesian | *ABCG2* | rs2231142 | Gout | 47.2 | 61.4 | 34.6 |
| Tu, 2016 [28] | Taiwan |  |  |  |  |  |  |  |  |  |
|  |  | Case-control | General | Asian | *ABCG2* | rs2231142 | HUA | 54.5 | 100 | 24.8 |
|  |  | Case-control | General | Asian | *SLC2A9* | rs1014290 | Gout | 54.5 | 100 | 24.9 |
| Zheng, 2016 [25] | China | Case-control | General | Asian | *ABCG2, SLC2A9* | rs2231142, rs3733591, rs6855911 | Gout | 44 | 77.7 |  |
| Cheng, 2017 [30] | Taiwan | Cohort | General | Asian | *ABCG2* | rs2231142, | Urate | 45 | 52.5 | 24.2 |
| Higashino, 2017 [21] | Japan | Case-control | General | Asian | *ABCG2* | rs2231142, rs2231137, rs72552713 | Gout | 49.3 | 100 | 24.3 |
| Kobylecki, 2017 [51] | Denmark | Cohort | General | Caucasian | *SLC2A9* | rs7442295 | Urate | 57.7 | 45 | 26 |
| Li, 2017 [24] | China | Cohort | General | Asian | *ABCG2, SLC2A9* | rs2231142, rs72552713, rs16890979 | Gout | 53.5 | 100 |  |
| Nakayama, 2017 [70] | Japan |  |  |  |  |  |  |  |  |  |
| - GWAS |  | Case-control | General | Asian | *SLC2A9* | rs1014290 | Gout | 57.4 | 100 | 23.3 |
| - Replication |  | Case-control | General | Asian | *SLC2A9* | rs1014290 | Gout | 46.5 | 100 | 24.1 |
| Stiburkova, 2017 [22] | Czech Republic | Cohort | General | Caucasian | *ABCG2* | rs2231142, rs2231137 | Gout | 55.5 | 90.3 | 29.5 |
| Yu, 2017 [23] | Taiwan | Case-control | General | Asian | *ABCG2* | rs2231142, rs2231137 | Gout | 52.3 | 92.5 |  |
| Chen, 2018 [20] | Taiwan |  |  |  |  |  |  |  |  |  |
|  |  | Case-control | General | Asian | *ABCG2* | rs2231142 | HUA | 49.6 | 100 | 25 |
|  |  | Case-control | General | Asian | *ABCG2* | rs2231142 | Gout | 49.9 | 100 | 25 |
| Tu, 2018 [50] | Taiwan | Case-control | General | Asian (Taiwanese Aborigine) | *SLC2A9* | rs1014290, rs37335591 | Gout | 52.7 | 62 | 26.3 |

*ABCG2*, ATP-binding cassette sub-family G member 2; ARIC, Atherosclerosis Risk in Communities; BMI, body mass index; CHD, coronary heart disease; CKD, chronic kidney disease; FHS, Framingham Heart Study; Go-DARTS, Genetics of Diabetes Audit and Research Tayside Study; GWAS, genome-wide association study; HUA, hyperuricemia; InCHIANTI, Invecchiare in Chianti, aging in the Chianti area; KORA, Kooperative Gesundheitsforschung in der Region Augsburg; NZ, New Zealand; SAPHIR, Salzburg Atherosclerosis Prevention Program in Subjects at High Individual Risk; SHIP, Study of Health in Pomerania; *SLC2A9*, solute carrier family 2 member 9; T2D, type 2 diabetes; UK, United Kingdom; USA, United States of America.
