# Supplementary material for: The association between genetic polymorphisms in ABCG2 and SLC2A9 and urate: an updated systematic review and meta-analysis
Source: BMC Med Genet. 2020 Oct 21;21:210. doi: 10.1186/s12881-020-01147-2 (PMC7580000; doi:10.1186/s12881-020-01147-2)
Supplement: Supplementary file 3 — Risk of bias assessment. (DOCX 26 kb) [file 12881_2020_1147_MOESM3_ESM.docx]

# Additional file 3. Risk of bias assessment

**A) Risk of bias assessment for dichotomous outcome (gout and hyperuricemia)**

| Author | Year | Information bias | | | Population stratification | Confounding bias | Selective outcome report | HWE |
| --- | --- | --- | --- | --- | --- | --- | --- | --- |
|  |  | Ascertainment of case | Ascertainment of control | Ascertainment of genotyping examination |  |  |  |  |
| Doring A. | 2008 |  |  |  |  |  |  |  |
| - KORA |  | Yes | Unclear | Yes | Yes | Yes | Yes | Yes |
| - SHIP |  | Yes | Unclear | Yes | Yes | Yes | Yes | Yes |
| Stark K. | 2008 | Yes | Yes | Yes | Yes | Yes | Yes | Yes |
| Vitart V. | 2008 |  |  |  |  |  |  |  |
| - Croatia |  | Unclear | Unclear | Yes | Yes | Yes | Yes | Unclear |
| - Germany |  | Unclear | Unclear | Unclear | Yes | Yes | No | Unclear |
| - Scotland   (Go-DARTS) |  | Yes | Yes | Unclear | Yes | Yes | No | Unclear |
| Hollis-Moffatt JE. | 2009 | Yes | Yes | Unclear | Yes | Yes | Yes | Yes |
| Matsuo H. | 2009 | Yes | Yes | Yes | Yes | Yes | Yes | Yes |
| Stark K. | 2009 | Yes | Yes | Yes | Yes | Yes | Yes | Yes |
| Woodward OM. | 2009 | Yes | Yes | Yes | Yes | Yes | Yes | Yes |
| Tu HP. | 2010 | Yes | No | Unclear | Yes | Yes | Yes | Yes |
| Urano W. | 2010 | Yes | No | Unclear | Yes | Yes | Yes | Yes |
| Wang B. | 2010 | Yes | Yes | Unclear | Yes | Yes | Yes | Yes |
| Yamagishi K. | 2010 | Yes | Yes | Yes | Yes | Yes | Yes | Yes |
| Guan M. | 2011 | Yes | Yes | Yes | Yes | No | Yes | Yes |
| Hollis-Moffatt JE. | 2011 |  |  |  |  |  |  |  |
| - ARIC |  | Yes | Yes | Unclear | Yes | No | Yes | Yes |
| - FHS |  | Yes | Yes | Unclear | Yes | No | Yes | Yes |
| - NZ |  | Yes | Yes | Unclear | Yes | No | Yes | Yes |
| Liu WC. | 2011 | Unclear | Unclear | Unclear | Yes | Yes | No | Yes |
| Takeuchi F. | 2013 | Yes | Yes | Yes | Yes | Yes | Yes | Yes |
| Urano W. | 2013 | Yes | No | Yes | Yes | Yes | Yes | Yes |
| Tu HP. | 2014 | Yes | Yes | Yes | Yes | Yes | Yes | Yes |
| Wang Q. | 2014 | Yes | Yes | Yes | Yes | No | Yes | Yes |
| Zhou D. | 2014 | Yes | Yes | Yes | Yes | Yes | Yes | Yes |
| Kim YS. | 2015 | Yes | Yes | Unclear | Yes | Yes | Yes | Yes |
| Mahfudzah A. | 2015 | Yes | Yes | Yes | Yes | No | Yes | Yes |
| Wan W. | 2015 | Yes | Yes | Unclear | Yes | Yes | Yes | Yes |
| Jiri M. | 2016 | Yes | Yes | Unclear | Yes | Yes | Yes | Yes |
| Kannangara DR. | 2016 | Yes | Yes | Yes | No | Yes | Yes | Yes |
| Matsuo H. | 2016 | Yes | Yes | Yes | Yes | Yes | No | Yes |
| Oetjens MT. | 2016 | Yes | Yes | Yes | Yes | Yes | No | Yes |
| Phipps-Green AJ. | 2016 | Yes | Yes | Yes | Yes | Yes | Yes | Yes |
| Tu HP. | 2016 | Yes | Yes | Yes | Yes | Yes | Yes | Yes |
| Zheng C. | 2016 | Yes | Yes | Unclear | Yes | Yes | Yes | Yes |
| Higashino T. | 2017 | Yes | Yes | Yes | Yes | Yes | Yes | Yes |
| Li Z. | 2017 | Yes | Yes | Yes | Yes | Yes | Yes | Yes |
| Nakayama A. | 2017 |  |  |  |  |  |  |  |
| - GWAS |  | Yes | Yes | Yes | Yes | Yes | Yes | Yes |
| - Replication |  | Yes | Yes | Yes | Yes | Yes | No | Yes |
| Stiburkova B. | 2017 | Yes | Yes | Yes | Yes | Yes | Yes | Yes |
| Yu KH. | 2017 | Yes | Yes | Yes | Yes | No | Yes | Yes |
| Chen CJ. | 2018 | Yes | Yes | Yes | Yes | Yes | No | Yes |
| Tu HP. | 2018 | Yes | Yes | Unclear | Yes | Yes | Yes | Yes |

ARIC, Atherosclerosis Risk in Communities; FHS, Framingham Heart Study; Go-DARTS, Genetics of Diabetes Audit and Research Tayside Study; GWAS, genome-wide association study; HWE, Hardy-Weinberg equilibrium; KORA, Kooperative Gesundheitsforschung in der Region Augsburg; NZ, New Zealand; SHIP, Study of Health in Pomerania.

1. **Risk of bias assessment for continuous outcome (serum urate)**

| Author | Year | Information bias | | Population stratification | Confounding bias | Selective outcome report | HWE |
| --- | --- | --- | --- | --- | --- | --- | --- |
|  |  | Ascertainment of outcome | Ascertainment of genotyping examination |  |  |  |  |
| Li S. | 2007 |  |  |  |  |  |  |
| - InCHIANTI |  | Yes | Yes | Yes | Yes | Yes | Unclear |
| - SardiNIA |  | Yes | Yes | Yes | Yes | Yes | Unclear |
| Brandstatter A. | 2008 |  |  |  |  |  |  |
| - Utah |  | Yes | Yes | Yes | Yes | Yes | Yes |
| Vitart V. | 2008 |  |  |  |  |  |  |
| - Scotland   (Orkney) |  | Unclear | Unclear | Yes | Yes | No | Unclear |
| Brandstatter A. | 2010 |  |  |  |  |  |  |
| - Bruneck |  | Yes | Yes | Yes | Yes | Yes | Yes |
| - SAPHIR |  | Yes | Yes | Yes | Yes | Yes | Yes |
| Cummings N. | 2010 | Unclear | Unclear | No | Yes | Yes | Yes |
| Tabara Y. | 2010 |  |  |  |  |  |  |
| - Ehime |  | Unclear | Yes | Yes | Yes | Yes | Unclear |
| - Suita |  | Unclear | Yes | Yes | Yes | Yes | Unclear |
| Hu M. | 2012 | Yes | Yes | Yes | No | Yes | Yes |
| Lyngdoh T. | 2012 | Yes | Yes | Yes | Yes | Yes | Yes |
| Voruganti VS. | 2013 | Yes | Yes | Yes | Yes | Yes | Yes |
| Stiburkova B. | 2014 | Unclear | Yes | Yes | Yes | Yes | Yes |
| Testa A. | 2014 | No | Yes | Yes | Yes | Yes | Yes |
| Laston SL. | 2015 | Unclear | Yes | Yes | Yes | Yes | Yes |
| Mallamaci F. | 2015 | Yes | Yes | Yes | Yes | Yes | Yes |
| Zhang XY. | 2015 | Yes | Unclear | Yes | No | Yes | Unclear |
| Bartakova V. | 2016 | Yes | Unclear | Yes | Yes | Yes | Yes |
| Cheng ST. | 2017 | Unclear | Unclear | Yes | Yes | Yes | Yes |
| Kobylecki CJ. | 2017 | Unclear | Yes | Yes | Yes | Yes | Yes |

HWE, Hardy-Weinberg equilibrium; InCHIANTI, Invecchiare in Chianti, aging in the Chianti area; SAPHIR, Salzburg Atherosclerosis Prevention Program in Subjects at High Individual Risk.
