# Supplementary material for: The association between genetic polymorphisms in ABCG2 and SLC2A9 and urate: an updated systematic review and meta-analysis
Source: BMC Med Genet. 2020 Oct 21;21:210. doi: 10.1186/s12881-020-01147-2 (PMC7580000; doi:10.1186/s12881-020-01147-2)
Supplement: Supplementary file 4 — Gout. 4.1. Data used for pooling effects of ABCG2 and SLC2A9 polymorphisms on gout. 4.2. Pooled prevalence of minor allele of ABCG2 and SLC2A9 polymorphisms. 4.3. Exploring source of heterogeneity for ABCG2 and SLC2A9 polymorphisms on gout.4.4. Egger’s tests for ABCG2 and SLC2A9 polymorphisms on gout. 4.5. Funnel plots for ABCG2 and SLC2A9 polymorphisms on gout. (DOCX 662 kb) [file 12881_2020_1147_MOESM4_ESM.docx]

# Additional file 4. Gout

## Additional file 4.1. Data used for pooling effects of *ABCG2* and *SLC2A9* polymorphisms on gout

| *ABCG2* rs2231142 | | | | | | | | | | | | | | | | | | | | | | | | | | | |  |
| --- | --- | --- | --- | --- | --- | --- | --- | --- | --- | --- | --- | --- | --- | --- | --- | --- | --- | --- | --- | --- | --- | --- | --- | --- | --- | --- | --- | --- |
| Author | | Year | Gout | | | | | | | | | | Control | | | | | | | HWE | | AA/CC | | | CA/CC | | |  |
|  |  |  | No. of subjects | Genotype | | | | | | | | | No. of subjects | Genotype | | | | | | p-value | | OR_1_ | 95% CI | | OR_2_ | | 95% CI |  |
|  |  |  |  | CC | | | CA | | | AA | | |  | CC | CA | | AA | | |  | |  |  |  |  |  |  |  |
| **Caucasians** | | | | | | | | | | | | | | | | | | | | | | | | | | | |  |
| Stark K. | | 2009 | 677 | 500 | | | 168 | | | 9 | | | 1552 | 1241 | 299 | | 12 | | | 0.191 | | 1.86 | 0.78, 4.45 | | 1.39 | | 1.12, 1.73 |  |
| Woodward OM. | | 2009 | 455 | 327 | | | 118 | | | 10 | | | 8034 | 6465 | 1483 | | 86 | | | 0.926 | | 2.30 | 1.18, 4.47 | | 1.57 | | 1.27, 1.96 |  |
| Oetjens MT. | | 2016 | 363 | 250 | | | 102 | | | 11 | | | 5528 | 4379 | 1082 | | 67 | | | 0.986 | | 2.88 | 1.50, 5.51 | | 1.65 | | 1.30, 2.10 |  |
| Phipps-Green AJ. | | 2016 | 638 | 392 | | | 209 | | | 37 | | | 1541 | 1169 | 352 | | 20 | | | 0.258 | | 5.52 | 3.16, 9.62 | | 1.77 | | 1.44, 2.18 |  |
| Stiburkova B. | | 2017 | 145 | 84 | | | 55 | | | 6 | | | 115 | 86 | 13 | | 1 | | | 0.433 | | 6.14 | 0.72, 52.12 | | 4.33 | | 2.21, 8.51 |  |
| Pooled OR | | | | | | | | | | | | | | | | | | | |  | | 3.24 | 2.39, 4.41 | | 1.64 | | 1.47, 1.82 |  |
| **Asians** | | | | | | | | | | | | | | | | | | | | | | | | | | | |  |
| Matsuo H.^a^ | | 2009 | 159 | 41 | | | 87 | | | 31 | | | 865 | 462 | 316 | | 87 | | | 0.003 | | 4.02 | 2.39, 6.75 | | 3.10 | | 2.08, 4.62 |  |
| Wang B. | | 2010 | 200 | 64 | | | 91 | | | 45 | | | 235 | 103 | 112 | | 20 | | | 0.172 | | 3.62 | 1.96, 6.68 | | 1.31 | | 0.86, 1.98 |  |
| Yamagishi K. | | 2010 | 45 | 15 | | | 18 | | | 12 | | | 3878 | 1846 | 1655 | | 377 | | | 0.829 | | 3.92 | 1.82, 8.44 | | 1.34 | | 0.67, 2.66 |  |
| Urano W. | | 2013 | 153 | 46 | | | 76 | | | 31 | | | 532 | 275 | 215 | | 42 | | | 0.998 | | 4.41 | 2.52, 7.72 | | 2.11 | | 1.41, 3.18 |  |
| Tu HP._1 | | 2014 | 254 | 61 | | | 104 | | | 89 | | | 446 | 217 | 180 | | 49 | | | 0.210 | | 6.46 | 4.12, 10.13 | | 2.06 | | 1.42, 2.98 |  |
| Tu HP._2 | | 2014 | 533 | 128 | | | 230 | | | 175 | | | 1105 | 364 | 518 | | 223 | | | 0.119 | | 2.23 | 1.68, 2.96 | | 1.26 | | 0.98, 1.63 |  |
| Wang Q. | | 2014 | 185 | 64 | | | 86 | | | 35 | | | 311 | 157 | 126 | | 28 | | | 0.707 | | 3.07 | 1.72, 5.45 | | 1.67 | | 1.12, 2.50 |  |
| Zhou D. | | 2014 | 352 | 87 | | | 181 | | | 84 | | | 350 | 167 | 150 | | 33 | | | 0.935 | | 4.89 | 3.03, 7.89 | | 2.32 | | 1.65, 3.25 |  |
| Kim YS. | | 2015 | 109 | 26 | | | 51 | | | 32 | | | 102 | 53 | 44 | | 5 | | | 0.275 | | 13.05 | 4.55, 37.39 | | 2.36 | | 1.27, 4.39 |  |
| Wan W. | | 2015 | 97 | 22 | | | 49 | | | 26 | | | 101 | 54 | 38 | | 9 | | | 0.539 | | 7.09 | 2.87, 17.54 | | 3.17 | | 1.65, 6.08 |  |
| Jiri M. | | 2016 | 143 | 71 | | | 59 | | | 12 | | | 310 | 154 | 129 | | 27 | | | 0.998 | | 0.96 | 0.46, 2.01 | | 0.99 | | 0.65, 1.51 |  |
| Matsuo H. | | 2016 | 1193 | 581 | | | 990 | | | 422 | | | 1334 | 711 | 526 | | 97 | | | 0.983 | | 5.32 | 4.16, 6.81 | | 2.30 | | 1.98, 2.68 |  |
| Zheng C. | | 2016 | 155 | 34 | | | 77 | | | 44 | | | 140 | 59 | 62 | | 19 | | | 0.674 | | 4.02 | 2.03, 7.96 | | 2.16 | | 1.26, 3.69 |  |
| Higashino T. | | 2017 | 477 | 152 | | | 233 | | | 92 | | | 480 | 262 | 185 | | 33 | | | 0.965 | | 4.81 | 3.08, 7.50 | | 2.17 | | 1.64, 2.87 |  |
| Li Z. | | 2017 | 1255 | 319 | | | 627 | | | 309 | | | 1848 | 882 | 789 | | 177 | | | 0.977 | | 4.83 | 3.86, 6.04 | | 2.20 | | 1.86, 2.59 |  |
| Yu KH. | | 2017 | 210 | 36 | | | 102 | | | 72 | | | 123 | 60 | 52 | | 11 | | | 0.956 | | 10.91 | 5.12, 23.26 | | 3.27 | | 1.92, 5.56 |  |
| Chen CJ. | | 2018 | 747 | 193 | | | 385 | | | 169 | | | 2070 | 1088 | 812 | | 170 | | | 0.287 | | 5.60 | 4.31, 7.28 | | 2.67 | | 2.20, 3.25 |  |
| Pooled OR | | | | | | | | | | | | | | | | | | | |  | | 4.53 | 4.10, 5.00 | | 2.10 | | 1.95, 2.26 |  |
| **Africans** | | | | | | | | | | | | | | | | | | | | | | | | | | | |  |
| Oetjens MT. | | 2016 | 64 | 60 | | | 4 | | | 0 | | | 617 | 580 | 36 | | 1 | | | 0.444 | | 3.20 | 0.13, 79.37 | | 1.07 | | 0.37, 3.12 |  |
| **Polynesians** | | | | | | | | | | | | | | | | | | | | | | | | | | | |  |
| Phipps-Green AJ. | | 2016 | 876 | 494 | | | 288 | | | 94 | | | 1081 | 824 | 229 | | 28 | | | 0.014 | | 5.60 | 3.62, 8.66 | | 2.10 | | 1.71, 2.58 |  |
| *ABCG2* rs72552713 | | | | | | | | | | | | | | | | | | | | | | | | | | | |  |
| Author | | Year | Gout | | | | | | | | | | Control | | | | | | | HWE | TT/CC | | | | CT/CC | | |  |
|  |  |  | No. of subjects | Genotype | | | | | | | | | No. of subjects | Genotype | | | | | | p-value | OR_1_ | | | 95% CI | OR_2_ | | 95% CI |  |
|  |  |  |  | CC | | | CT | | | TT | | |  | CC | CT | | TT | | |  |  |  |  |  |  |  |  |  |
| **Asians** | | | | | | | | | | | | | | | | | | | | | | | | | | | |  |
| Matsuo H. | | 2009 | 161 | 139 | | | 21 | | | 1 | | | 871 | 840 | 31 | | 0 | | | 1.000 | 18.08 | | | 0.73, 445.93 | 4.11 | | 2.31, 7.32 |  |
| Urano W. | | 2013 | 153 | 138 | | | 15 | | | 0 | | | 532 | 511 | 21 | | 0 | | | 1.000 | 3.69 | | | 0.07, 186.96 | 2.66 | | 1.35, 5.25 |  |
| Tu HP._1 | | 2014 | 254 | 250 | | | 4 | | | 0 | | | 446 | 445 | 1 | | 0 | | | 1.000 | 1.78 | | | 0.04, 89.90 | 5.34 | | 0.84, 34.08 |  |
| Tu HP._2 | | 2014 | 533 | 533 | | | 0 | | | 0 | | | 1105 | 1105 | 0 | | 0 | | | 1.000 | 2.07 | | | 0.04, 104.57 | 2.07 | | 0.04, 104.57 |  |
| Zhou D. | | 2014 | 352 | 319 | | | 33 | | | 0 | | | 350 | 338 | 12 | | 0 | | | 1.000 | 1.06 | | | 0.02, 53.55 | 2.84 | | 1.46, 5.53 |  |
| Matsuo H. | | 2016 | 1993 | 1802 | | | 186 | | | 5 | | | 1334 | 1273 | 60 | | 1 | | | 0.516 | 3.53 | | | 0.41, 30.27 | 2.19 | | 1.62, 2.96 |  |
| Higashino T. | | 2017 | 478 | 423 | | | 53 | | | 2 | | | 478 | 455 | 23 | | 0 | | | 1.000 | 5.38 | | | 0.26, 112.34 | 2.45 | | 1.48, 4.05 |  |
| Li Z. | | 2017 | 1255 | 1233 | | | 22 | | | 0 | | | 1848 | 1830 | 18 | | 0 | | | 1.000 | 1.48 | | | 0.03, 74.84 | 1.80 | | 0.97, 3.35 |  |
| Pooled OR | | | | | | | | | | | | | | | | | | | |  | 3.86 | | | 2.30, 9.76 | 2.46 | | 1.93, 3.18 |  |
|  | *ABCG2* rs2231137 | | | | | | | | | | | | | | | | | | | | | | | | | | | |
| Author | | Year | Gout | | | | | | | | Control | | | | | | | | | HWE | | AA/GG | | | | GA/GG | | |
|  |  |  | No. of subjects | Genotype | | | | | | | No. of subjects | | | Genotype | | | | | | p-value | | OR_1_ | 95% CI | | | OR_2_ | 95% CI | |
|  |  |  |  | GG | GA | | | AA | | |  |  |  | GG | | GA | | AA | |  |  |  |  |  |  |  |  |  |
| **Asians** | | | | | | | | | | | | | | | | | | | | | | | | | | | | |
| Matsuo H. | | 2009 | 158 | 112 | | 43 | | | 3 | | | 862 | | 526 | | 306 | | | 30 | 0.072 | | 0.47 | 0.14, 1.57 | | | 0.66 | 0.45, 0.96 | |
| Tu HP._1 | | 2014 | 254 | 131 | 101 | | | 22 | | | 446 | | | 199 | | 184 | | | 63 | 0.057 | | 0.53 | 0.31, 0.90 | | | 0.83 | 0.60, 1.16 | |
| Tu HP._2 | | 2014 | 533 | 299 | 195 | | | 39 | | | 1105 | | | 518 | | 471 | | | 116 | 0.561 | | 0.58 | 0.39, 0.86 | | | 0.72 | 0.58, 0.89 | |
| Zhou D. | | 2014 | 352 | 239 | 97 | | | 16 | | | 350 | | | 182 | | 133 | | | 35 | 0.149 | | 0.35 | 0.19, 0.65 | | | 0.56 | 0.40, 0.77 | |
| Higashino T. | | 2017 | 479 | 361 | 109 | | | 9 | | | 479 | | | 299 | | 159 | | | 21 | 0.981 | | 0.35 | 0.16, 0.79 | | | 0.57 | 0.43, 0.76 | |
| Yu KH. | | 2017 | 210 | 143 | 61 | | | 6 | | | 123 | | | 47 | | 58 | | | 18 | 0.988 | | 0.11 | 0.04, 0.29 | | | 0.35 | 0.16, 0.79 | |
| Pooled OR | | | | | | | | | | | | | | | | | | | |  | | 0.43 | 0.34, 0.55 | | | 0.64 | 0.56, 0.72 | |
| **Caucasians** | | | | | | | | | | | | | | | | | | | | | | | | | | | | |
| Stiburkova B. | | 2017 | 145 | 138 | 7 | | | 0 | | | 100 | | | 97 | | 3 | | | 0 | 1.000 | | 0.70 | 0.01, 35.78 | | 1.51 | | 0.41, 5.51 | |

|  | *SLC2A9* rs1014290 | | | | | | | | | | | | | | |
| --- | --- | --- | --- | --- | --- | --- | --- | --- | --- | --- | --- | --- | --- | --- | --- |
| Author | | Year | Gout | | | | Control | | | | HWE | CC/TT | | TC/TT | |
|  |  |  | No. of subjects | Genotype | | | No. of subjects | Genotype | | | p-value | OR_1_ | 95% CI | OR_2_ | 95% CI |
|  |  |  |  | TT | TC | CC |  | TT | TC | CC |  |  |  |  |  |
| **Caucasians** | | | | | | | | | | | | | | | |
| Vitart V. | | 2008 |  |  |  |  |  |  |  |  |  |  |  |  |  |
| - Croatia | |  | 57 | 31 | 22 | 4 | 440 | 210 | 188 | 42 | 0.994 | 0.65 | 0.22, 1.92 | 0.79 | 0.44, 1.42 |
| - Germany | | 2008 | 148 | 97 | 46 | 5 | 231 | 137 | 82 | 12 | 0.953 | 0.59 | 0.20, 1.73 | 0.79 | 0.51, 1.24 |
| - Scotland   (Go-DARTS) | | 2008 | 484 | 325 | 143 | 16 | 9659 | 5727 | 3421 | 511 | 0.997 | 0.55 | 0.33, 0.92 | 0.74 | 0.60, 0.90 |
| Pooled OR | | | | | | | | | | |  | 0.57 | 0.37, 0.87 | 0.75 | 0.63, 0.89 |
| **Asians** | | | | | | | | | | | | | | | |
| Urano W. | | 2010 | 177 | 90 | 72 | 15 | 577 | 204 | 279 | 94 | 0.932 | 0.36 | 0.20, 0.66 | 0.58 | 0.41, 0.84 |
| Liu WC. | | 2011 | 61 | 28 | 24 | 9 | 942 | 296 | 489 | 157 | 0.060 | 0.61 | 0.28, 1.32 | 0.52 | 0.30, 0.89 |
| Tu HP. | | 2016 | 157 | 82 | 66 | 9 | 295 | 101 | 138 | 56 | 0.469 | 0.20 | 0.09, 0.42 | 0.59 | 0.39, 0.89 |
| Nakayama A. | | 2017 |  |  |  |  |  |  |  |  |  |  |  |  |  |
| - GWAS | |  | 945 | 434 | 413 | 98 | 1213 | 386 | 596 | 231 | 0.972 | 0.38 | 0.29, 0.50 | 0.62 | 0.51, 0.74 |
| - Replica-tion | |  | 1396 | 632 | 615 | 149 | 1268 | 421 | 619 | 228 | 0.986 | 0.44 | 0.34, 0.55 | 0.66 | 0.56, 0.78 |
| Tu HP. | | 2018 | 373 | 181 | 158 | 34 | 305 | 136 | 137 | 32 | 0.773 | 0.80 | 0.47, 1.36 | 0.87 | 0.63, 1.19 |
| Pooled OR | | | | | | | | | | |  | 0.42 | 0.36, 0.49 | 0.65 | 0.58, 0.72 |
| **Solomon Islanders** | | | | | | | | | | |  |  |  |  |  |
| Tu HP. | | 2010 | 69 | 31 | 32 | 6 | 168 | 68 | 78 | 22 | 0.960 | 0.60 | 0.22, 1.62 | 0.90 | 0.50, 1.63 |

| *SLC2A9* rs3733591 | | | | | | | | | | | | | | |
| --- | --- | --- | --- | --- | --- | --- | --- | --- | --- | --- | --- | --- | --- | --- |
| Author | Year | Gout | | | | Control | | | | HWE | CC/TT | | TC/TT | |
|  |  | No. of subjects | Genotype | | | No. of subjects | Genotype | | | p-value | OR_1_ | 95% CI | OR_2_ | 95% CI |
|  |  |  | TT | TC | CC |  | TT | TC | CC |  |  |  |  |  |
| **Caucasians** | | | | | | | | | | | | | | |
| Hollis-Moffatt JE. | 2011 |  |  |  |  |  |  |  |  |  |  |  |  |  |
| - ARIC |  | 153 | 6 | 30 | 117 | 6968 | 168 | 1898 | 4902 | 0.325 | 0.67 | 0.29, 1.54 | 0.44 | 0.18, 1.08 |
| - FHS |  | 67 | 2 | 21 | 44 | 4712 | 169 | 1385 | 3158 | 0.263 | 1.18 | 0.28, 4.90 | 1.28 | 0.30, 5.51 |
| - NZ |  | 313 | 9 | 87 | 217 | 636 | 23 | 196 | 417 | 0.996 | 1.33 | 0.60, 2.92 | 1.13 | 0.50, 2.55 |
| Pooled OR | | | | | | | | | |  | 1.03 | 0.60, 1.79 | 0.85 | 0.48, 1.50 |
| **Asians** | | | | | | | | | | | | | | |
| Tu HP. | 2010 | 109 | 35 | 50 | 24 | 191 | 85 | 91 | 15 | 0.164 | 3.89 | 1.83, 8.27 | 1.33 | 0.79, 2.25 |
| Urano W. | 2010 | 178 | 58 | 93 | 27 | 576 | 268 | 252 | 56 | 0.771 | 2.23 | 1.30, 3.82 | 1.71 | 1.18, 2.47 |
| Mahfudzah A. | 2015 | 82 | 69 | 12 | 1 | 78 | 74 | 4 | 0 | 1.000 | 3.22 | 0.13, 80.27 | 3.22 | 0.99, 10.45 |
| Wan W. | 2015 | 97 | 36 | 49 | 12 | 100 | 45 | 46 | 9 | 0.569 | 1.67 | 0.63, 4.39 | 1.33 | 0.73, 2.41 |
| Zheng C. | 2016 | 163 | 42 | 95 | 26 | 187 | 61 | 99 | 27 | 0.194 | 1.40 | 0.72, 2.72 | 1.39 | 0.86, 2.26 |
| Tu HP. | 2018 | 373 | 150 | 183 | 40 | 305 | 113 | 152 | 40 | 0.317 | 0.75 | 0.46, 1.24 | 0.91 | 0.66, 1.26 |
| Pooled OR | | | | | | | | | |  | 1.55 | 1.17, 2.06 | 1.29 | 1.07, 1.55 |
| **Solomon Islanders** | | | | | | | | | | | | | | |
| Tu HP. | 2010 | 69 | 23 | 28 | 18 | 168 | 62 | 75 | 31 | 0.327 | 1.57 | 0.74, 3.32 | 1.01 | 0.53, 1.92 |
| **Polynesians** | | | | | | | | | | | | | | |
| Hollis-Moffatt JE. | 2011 | 606 | 84 | 263 | 259 | 800 | 100 | 334 | 366 | 0.083 | 0.84 | 0.61, 1.17 | 0.94 | 0.67, 1.31 |

| *SLC2A9* rs6449213 | | | | | | | | | | | | | | | | | | | | | | | | | | | |
| --- | --- | --- | --- | --- | --- | --- | --- | --- | --- | --- | --- | --- | --- | --- | --- | --- | --- | --- | --- | --- | --- | --- | --- | --- | --- | --- | --- |
| Author | | Year | | | | Gout | | | | | | | | Control | | | | | | | | HWE | | CC/TT | | TC/TT | |
|  |  |  |  |  |  | No. of subjects | | Genotype | | | | | | No. of subjects | | Genotype | | | | | | p-value | | OR_1_ | 95% CI | OR_2_ | 95% CI |
|  |  |  |  |  |  |  |  | TT | | TC | | CC | |  |  | TT | | TC | | CC | |  | |  |  |  |  |
| **Caucasians** | | | | | | | | | | | | | | | | | | | | | | | | | | | |
| Doring A. | | 2008 | | | |  | |  | |  | |  | |  | |  | |  | |  | |  | |  |  |  |  |
| - KORA | |  | | | | 557 | | 305 | | 214 | | 38 | | 5249 | | 2110 | | 2436 | | 703 | | 0.998 | | 0.37 | 0.26, 0.53 | 0.61 | 0.51, 0.73 |
| Vitart V. | | 2008 | | | |  | |  | |  | |  | |  | |  | |  | |  | |  | |  |  |  |  |
| - Croatia | |  | | | | 57 | | 35 | | 19 | | 3 | | 440 | | 248 | | 165 | | 27 | | 0.949 | | 0.79 | 0.23, 2.73 | 0.82 | 0.45, 1.48 |
| - Germany | |  | | | | 148 | | 112 | | 33 | | 3 | | 231 | | 152 | | 71 | | 8 | | 0.934 | | 0.51 | 0.13, 1.96 | 0.63 | 0.39, 1.02 |
| - Scotland   (Go-DARTS) | |  | | | | 484 | | 358 | | 117 | | 9 | | 9659 | | 6495 | | 2851 | | 313 | | 0.995 | | 0.52 | 0.27, 1.02 | 0.74 | 0.60, 0.92 |
| Stark K. | | 2008 | | | | 656 | | 469 | | 181 | | 6 | | 660 | | 421 | | 207 | | 32 | | 0.319 | | 0.17 | 0.07, 0.41 | 0.78 | 0.62, 1.00 |
| Pooled OR | | | | | | | | | | | | | | | | | | | | | |  | | 0.38 | 0.29, 0.50 | 0.69 | 0.62, 0.78 |
| **Asians** | | | | | | | | | | | | | | | | | | | | | | | | | | | |
| Urano W. | | 2010 | | | | 179 | | 176 | | 3 | | 0 | | 576 | | 566 | | 9 | | 1 | | 0.047 | | 1.07 | 0.04, 26.38 | 1.18 | 0.34, 4.07 |
| Kim YS. | | 2015 | | | | 109 | | 107 | | 2 | | 0 | | 102 | | 101 | | 1 | | 0 | | 1.000 | | 0.94 | 0.02, 48.03 | 1.57 | 0.20, 12.13 |
| *SLC2A9* rs16890979 | | | | | | | | | | | | | | | | | | | | | | | | | | | |
| Author | | | | Year | | | Gout | | | | | | | | Control | | | | | | | | HWE | TT/CC | | CT/CC | |
|  |  |  |  |  |  |  | No. of subjects | | Genotype | | | | | | No. of subjects | | Genotype | | | | | | p-value | OR_1_ | 95% CI | OR_2_ | 95% CI |
|  |  |  |  |  |  |  |  |  | CC | | CT | | TT | |  |  | CC | | CT | | TT | |  |  |  |  |  |
| **Caucasians** | | | | | | | | | | | | | | | | | | | | | | | | | | | |
| Hollis-Moffatt JE. | | | 2009 | | | | 131 | | 103 | | 24 | | 4 | | 551 | | 338 | | 183 | | 30 | | 0.427 | 0.44 | 0.15, 1.27 | 0.43 | 0.27, 0.70 |
| **Asians** | | | | | | | | | | | | | | | | | | | | | | | | | | | |
| Urano W. | | | | 2010 | | | 180 | | 178 | | 2 | | 0 | | 591 | | 580 | | 11 | | 0 | | 1.000 | 3.25 | 0.06, 164.48 | 0.71 | 0.18, 2.80 |
| Kim YS. | | | | 2015 | | | 109 | | 109 | | 0 | | 0 | | 102 | | 100 | | 2 | | 0 | | 1.000 | 0.92 | 0.02, 46.69 | 0.18 | 0.01, 3.87 |
| Li Z. | | | | 2017 | | | 1255 | | 1228 | | 27 | | 0 | | 1848 | | 1785 | | 62 | | 1 | | 0.426 | 0.48 | 0.02, 11.90 | 0.64 | 0.41, 1.01 |
| Pooled OR | | | | | | | | | | | | | | | | | | | | | | |  | NA | NA | 0.60 | 0.39, 0.94 |
| **Polynesians** | | | | | | | | | | | | | | | | | | | | | | | | | | | |
| Hollis-Moffatt JE. | | | | 2009 | | | 123 | | 121 | | 2 | | 0 | | 157 | | 118 | | 35 | | 4 | | 0.494 | 0.11 | 0.01, 2.04 | 0.07 | 0.02, 0.25 |
| *SLC2A9* rs6855911 | | | | | | | | | | | | | | | | | | | | | | | | | | | |
| Author | Year | | | | Gout | | | | | | | | | | Control | | | | | | | | HWE | GG/AA | | AG/AA | |
|  |  |  |  |  | No. of subjects | | | | Genotype | | | | | | No. of subjects | | Genotype | | | | | | p-value | OR_1_ | 95% CI | OR_2_ | 95% CI |
|  |  |  |  |  |  |  |  |  | AA | | AG | | GG | |  |  | AA | | AG | | GG | |  |  |  |  |  |
| **Caucasians** | | | | | | | | | | | | | | | | | | | | | | | | | | | |
| Doring A. | 2008 | | | |  | | | |  | |  | |  | |  | |  | |  | |  | |  |  |  |  |  |
| - KORA | | |  | | | | 557 | | 244 | | 249 | | 64 | | 5249 | | 1571 | | 2601 | | 1077 | | 0.994 | 0.38 | 0.29, 0.51 | 0.62 | 0.51, 0.74 |
| Stark K. | 2009 | | | | 677 | | | | 429 | | 233 | | 15 | | 1546 | | 829 | | 603 | | 114 | | 0.763 | 0.25 | 0.15, 0.44 | 0.75 | 0.62, 0.90 |
| Pooled OR | | | | | | | | | | | | | | | | | | | | | | |  | 0.36 | 0.28, 0.46 | 0.67 | 0.59, 0.77 |
| **Asians** | | | | | | | | | | | | | | | | | | | | | | | | | | | |
| Urano W. | 2010 | | | | 179 | | | | 177 | | 2 | | 0 | | 581 | | 570 | | 11 | | 0 | | 1.000 | 3.21 | 0.06, 162.57 | 0.70 | 0.18, 2.77 |
| Guan M. | 2011 | | | | 166 | | | | 160 | | 6 | | 0 | | 206 | | 197 | | 9 | | 0 | | 1.000 | 1.23 | 0.02, 62.36 | 0.84 | 0.30, 2.33 |
| Zheng C. | 2016 | | | | 163 | | | | 125 | | 33 | | 5 | | 187 | | 135 | | 43 | | 9 | | 0.055 | 0.60 | 0.20, 1.84 | 0.83 | 0.50, 1.39 |
| Pooled OR | | | | | | | | | | | | | | | | | | | | | | |  | 0.61 | 0.20, 1.87 | 0.82 | 0.53, 1.27 |

| *SLC2A9* rs7442295 | | | | | | | | | | | | | | |
| --- | --- | --- | --- | --- | --- | --- | --- | --- | --- | --- | --- | --- | --- | --- |
| Author | Year | Gout | | | | Control | | | | HWE | GG/AA | | AG/AA | |
|  |  | No. of subjects | Genotype | | | No. of subjects | Genotype | | | p-value | OR_1_ | 95% CI | OR_2_ | 95% CI |
|  |  |  | AA | AG | GG |  | AA | AG | GG |  |  |  |  |  |
| **Caucasians** | | | | | | | | | | | | | | |
| Stark K. | 2008 | 658 | 449 | 200 | 9 | 659 | 389 | 230 | 40 | 0.443 | 0.19 | 0.09, 0.41 | 0.75 | 0.60, 0.95 |
| Doring A. | 2008 |  |  |  |  |  |  |  |  |  |  |  |  |  |
| - KORA |  | 557 | 274 | 233 | 50 | 5249 | 1877 | 2524 | 848 | 0.992 | 0.40 | 0.30, 0.55 | 0.63 | 0.53, 0.76 |
| - SHIP |  | 260 | 140 | 102 | 18 | 3806 | 1449 | 1799 | 558 | 0.992 | 0.33 | 0.20, 0.55 | 0.59 | 0.45, 0.76 |
| Pooled OR | | | | | | | | | |  | 0.35 | 0.28, 0.46 | 0.65 | 0.57, 0.74 |
| **Asians** | | | | | | | | | | | | | | |
| Urano W. | 2010 | 177 | 176 | 1 | 0 | 587 | 578 | 9 | 0 | 1.000 | 3.28 | 0.06, 165.78 | 0.52 | 0.09, 2.92 |
| *SLC2A9* rs12510549 | | | | | | | | | | | | | | |
| Author | Year | Gout | | | | Control | | | | HWE | CC/TT | | TC/TT | |
|  |  | No. of subjects | Genotype | | | No. of subjects | Genotype | | | p-value | OR_1_ | 95% CI | OR_2_ | 95% CI |
|  |  |  | TT | TC | CC |  | TT | TC | CC |  |  |  |  |  |
| **Caucasians** | | | | | | | | | | | | | | |
| Doring A. | 2008 |  |  |  |  |  |  |  |  |  |  |  |  |  |
| - KORA |  | 557 | 264 | 239 | 54 | 5249 | 1896 | 2517 | 836 | 0.989 | 0.46 | 0.34, 0.63 | 0.68 | 0.57, 0.82 |
| Stark K. | 2008 | 660 | 459 | 192 | 9 | 656 | 392 | 238 | 26 | 0.170 | 0.30 | 0.14, 0.64 | 0.69 | 0.55, 0.87 |
| Hollis-Moffatt JE. | 2009 | 130 | 100 | 26 | 4 | 563 | 350 | 192 | 21 | 0.396 | 0.67 | 0.22, 1.99 | 0.47 | 0.30, 0.76 |
| Pooled OR | | | | | | | | | |  | 0.44 | 0.34, 0.58 | 0.66 | 0.58, 0.76 |
| **Polynesians** | | | | | | | | | | | | | | |
| Hollis-Moffatt JE. | 2009 | 119 | 112 | 7 | 0 | 163 | 141 | 22 | 0 | 1.000 | 1.26 | 0.02, 63.89 | 0.42 | 0.18, 0.99 |

^a^ Not included in pooling due to non-compliance with HWE.

*ABCG2*, ATP-binding cassette sub-family G member 2; ARIC, Atherosclerosis Risk in Communities; CI, confidence interval; FHS, Framingham Heart Study; Go-DARTS, Genetics of Diabetes Audit and Research Tayside Study; GWAS, genome-wide association study; KORA, Kooperative Gesundheitsforschung in der Region Augsburg; NZ, New Zealand; OR, odds ratio; SHIP, Study of Health in Pomerania; *SLC2A9*, solute carrier family 2 member 9.

## Additional file 4.2. Pooled prevalence of minor allele of *ABCG2* and *SLC2A9* polymorphisms

| SNP | Minor/Major allele | Ethnicity | Minor allele prevalence | 95% CI |
| --- | --- | --- | --- | --- |
| *ABCG2* |  |  |  |  |
| rs2231142 | A/C | Caucasian | 0.11 | 0.10, 0.12 |
|  |  | Asian | 0.31 | 0.28, 0.33 |
| rs72552713 | T/C | Asian | 0.015 | 0.008, 0.022 |
| rs2231137 | A/G | Asian | 0.29 | 0.24, 0.34 |
| *SLC2A9* |  |  |  |  |
| rs1014290 | C/T | Caucasian | 0.26 | 0.21, 0.31 |
|  |  | Asian | 0.41 | 0.39, 0.43 |
| rs3733591^a^ | T/C | Caucasian | 0.18 | 0.16, 0.20 |
|  | C/T | Asian | 0.29 | 0.15, 0.43 |
| rs6449213 | C/T | Caucasian | 0.24 | 0.14, 0.34 |
| rs6855911 | G/A | Caucasian | 0.40 | 0.39, 0.41 |
|  |  | Asian | 0.06 | 0.01, 0.11 |
| rs16890979 | T/C | Asian | 0.013 | 0.007, 0.019 |
| rs7442295 | G/A | Caucasian | 0.34 | 0.27, 0.41 |
| rs12510549 | C/T | Caucasian | 0.28 | 0.13, 0.42 |

^a^ For rs3733591, major allele in Caucasians is C whereas major allele in Asians is T.

*ABCG2*, ATP-binding cassette sub-family G member 2; CI, confidence interval; *SLC2A9*, solute carrier family 2 member 9; SNP, single nucleotide polymorphism.

## Additional file 4.3. Exploring source of heterogeneity for *ABCG2* and *SLC2A9* polymorphisms on gout

|  | No. of  sub-studies | OR_1_ | | | OR_2_ | | |
| --- | --- | --- | --- | --- | --- | --- | --- |
|  |  | OR | 95% CI | I^2^ (%) | OR | 95% CI | I^2^ (%) |
| ***ABCG2*** | | | | | | | |
| rs2231142 (Caucasians) | | | | | | | |
| Overall | 5 | 3.24 | 2.39, 4.41 | 39.2 | 1.64 | 1.47, 1.82 | 62.9 |
| Source of heterogeneity |  |  |  |  |  |  |  |
| Age | 4 | 3.36 | 2.37, 4.78 | 47.9 | 1.63 | 1.45, 1.84 | 81.4 |
| BMI | 4 | 3.36 | 2.37, 4.78 | 0.0 | 1.64 | 1.45, 1.84 | 44.1 |
| Percent male | 4 | 3.36 | 2.36, 4.78 | 23.1 | 1.64 | 1.45, 1.84 | 70.0 |
| Percent male^a^ | 4 | 3.36 | 2.37, 4.78 | 67.1 | 1.64 | 1.45, 1.84 | 18.9 |
| Subgroup analysis |  |  |  |  |  |  |  |
| %male < 90 | 3 | 3.29 | 2.30, 4.71 | 67.1 | 1.58 | 1.39, 1.78 | 18.9 |
| %male ≥ 90 | 1 | 6.14 | 0.72, 52.13 | NA | 4.33 | 2.21, 8.51 | NA |
| rs2231142 (Asians) | | | | | | | |
| Overall | 16 | 4.53 | 4.10, 5.00 | 75.2 | 2.10 | 1.95, 2.26 | 68.7 |
| Source of heterogeneity |  |  |  |  |  |  |  |
| Age | 16 | 4.53 | 4.10, 5.00 | 76.9 | 2.10 | 1.95, 2.26 | 70.6 |
| BMI | 9 | 4.51 | 3.99, 5.10 | 77.7 | 2.11 | 1.93, 2.30 | 72.6 |
| Percent male^a^ | 16 | 4.53 | 4.10, 5.00 | 33.6 | 2.10 | 1.95, 2.26 | 27.3 |
| Subgroup analysis |  |  |  |  |  |  |  |
| %male < 90 | 5 | 2.40 | 1.94, 2.97 | 62.3 | 1.35 | 1.14, 1.61 | 37.3 |
| %male ≥ 90 | 11 | 5.32 | 4.75, 5.97 | 4.6 | 2.29 | 2.11, 2.48 | 22.4 |
| rs2231137 (Asians) | | | | | | | |
| Overall | 6 | 0.43 | 0.34, 0.55 | 54.6 | 0.64 | 0.56, 0.72 | 54.9 |
| Source of heterogeneity |  |  |  |  |  |  |  |
| Age | 5 | 0.42 | 0.33, 0.55 | 72.0 | 0.63 | 0.55, 0.72 | 72.8 |
| Percent male^a^ | 6 | 0.43 | 0.33, 0.55 | 49.1 | 0.64 | 0.56, 0.72 | 57.4 |
| ***SLC2A9*** | | | | | | | |
| rs1014290 (Asians) | | | | | | | |
| Overall | 6 | 0.42 | 0.36, 0.49 | 55.0 | 0.65 | 0.58, 0.72 | 0.0 |
| Source of heterogeneity |  |  |  |  |  |  |  |
| Age | 5 | 0.43 | 0.36, 0.50 | 69.9 | NA |  | NA |
| BMI | 5 | 0.43 | 0.36, 0.50 | 57.6 | NA |  | NA |
| Percent male^a^ | 6 | 0.42 | 0.36, 0.49 | 6.8 | NA |  | NA |
| Subgroup analysis |  |  |  |  |  |  |  |
| %male < 100 | 2 | 0.75 | 0.48, 1.16 | 0.0 | NA |  | NA |
| %male = 100 | 4 | 0.39 | 0.33, 0.46 | 24.3 | NA |  | NA |
| rs3733591 (Asians) | | | | | | | |
| Overall | 6 | 1.55 | 1.17, 2.06 | 68.1 | 1.29 | 1.07, 1.55 | 45.2 |
| Source of heterogeneity |  |  |  |  |  |  |  |
| Age | 4 |  |  | 84.2 | NA^b^ |  | NA^b^ |
| Percent male^a^ | 6 | 1.54 | 1.16, 2.05 | 6.8 | 1.28 | 1.06, 1.54 | 8.4 |
| Subgroup analysis |  |  |  |  |  |  |  |
| %male < 90 | 2 | 0.92 | 0.62, 1.37 | 52.7 | 1.02 | 0.78, 1.34 | 52.1 |
| %male ≥ 90 | 4 | 2.52 | 1.71, 3.73 | 0.0 | 1.56 | 1.20, 2.03 | 0.0 |
| rs7442295 (Caucasians) | | | | | | | |
| Overall | 3 | 0.35 | 0.28, 0.46 | 38.7 | 0.65 | 0.57, 0.74 | 8.6 |
| Source of heterogeneity |  |  |  |  |  |  |  |
| Age | 3 | 0.35 | 0.28, 0.45 | 46.0 | NA |  | NA |
| Percent male^a^ | 3 | 0.36 | 0.28, 0.46 | 0.0 | NA |  | NA |
| Subgroup analysis |  |  |  |  |  |  |  |
| %male < 70 | 2 | 0.38 | 0.09, 0.50 | 0.0 | NA |  | NA |
| %male ≥ 70 | 1 | 0.19 | 0.09, 0.41 | NA | NA |  | NA |

^a^ Categorized variables, ^b^ No heterogeneity among studies with data of age available.

*ABCG2*, ATP-binding cassette sub-family G member 2; BMI, body mass index; CI, confidence interval; NA, not applicable; OR, odds ratio; *SLC2A9*, solute carrier family 2 member 9.

## Additional file 4.4. Egger’s tests for *ABCG2* and *SLC2A9* polymorphisms on gout

|  | No. of sub-studies | OR_1_ | | | OR_2_ | | |
| --- | --- | --- | --- | --- | --- | --- | --- |
|  |  | Coef. of Egger’s test | SE | *P* value | Coef. of Egger’s test | SE | *P* value |
| ***ABCG2*** |  |  |  |  |  |  |  |
| rs2231142 |  |  |  |  |  |  |  |
| - Caucasians | 5 | -0.45 | 1.95 | 0.834 | 4.14 | 1.47 | 0.066 |
| - Asians | 16 | 0.02 | 1.16 | 0.986 | -1.00 | 0.99 | 0.329 |
| rs72552713 |  |  |  |  |  |  |  |
| - Asians | 8 | -0.76 | 0.80 | 0.380 | 0.65 | 0.64 | 0.350 |
| rs2231137 |  |  |  |  |  |  |  |
| - Asians | 6 | -2.66 | 1.36 | 0.122 | -3.49 | 2.25 | 0.196 |
| ***SLC2A9*** |  |  |  |  |  |  |  |
| rs1014290 |  |  |  |  |  |  |  |
| - Caucasians | 3 | 0.38 | 0.18 | 0.285 | 0.46 | 0.12 | 0.157 |
| - Asians | 6 | 0.13 | 1.54 | 0.936 | -0.35 | 1.09 | 0.766 |
| rs3733591 |  |  |  |  |  |  |  |
| - Caucasians | 3 | 0.34 | 2.97 | 0.927 | 1.05 | 4.13 | 0.841 |
| - Asians | 6 | 1.45 | 1.97 | 0.503 | 2.29 | 1.48 | 0.195 |
| rs6449213 |  |  |  |  |  |  |  |
| - Caucasians | 5 | 0.37 | 1.25 | 0.787 | 0.65 | 1.29 | 0.648 |
| rs16890979 |  |  |  |  |  |  |  |
| - Asians | 3 | 3.42 | 2.63 | 0.417 | -0.53 | 0.58 | 0.530 |
| rs6855911 |  |  |  |  |  |  |  |
| - Asians | 3 | 0.84 | 0.36 | 0.260 | -0.24 | 0.25 | 0.519 |
| rs7442295 |  |  |  |  |  |  |  |
| - Caucasians | 3 | -3.07 | 0.73 | 0.149 | 0.01 | 5.68 | 0.999 |
| rs12510549 |  |  |  |  |  |  |  |
| - Caucasians | 3 | -0.17 | 1.51 | 0.926 | -2.48 | 0.87 | 0.215 |

*ABCG2*, ATP-binding cassette sub-family G member 2; Coef., coefficient; OR, odds ratio; SE, standard error; *SLC2A9*, solute carrier family 2 member 9.

## Additional file 4.5. Funnel plots for *ABCG2* and *SLC2A9* polymorphisms on gout

### Additional file 4.5.1. Funnel plots of rs2231142 on gout in Asians and Caucasians. A) OR_1_ in Asians B) OR_2_ in Asians C) OR_1_ in Caucasians D) OR_2_ in Caucasians


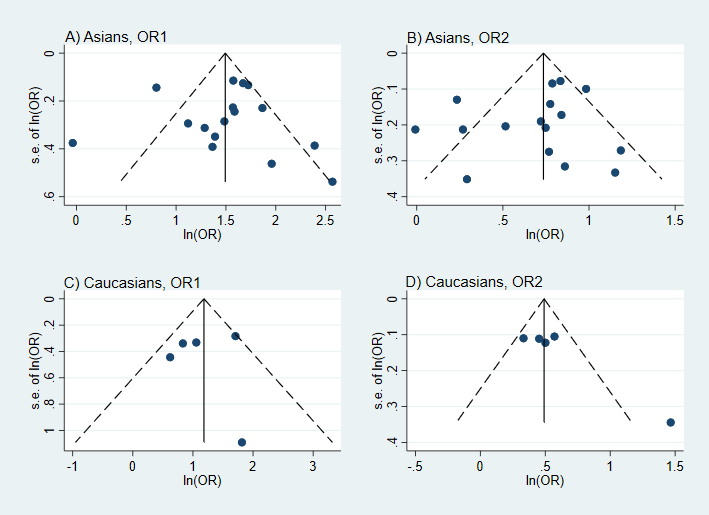


### **Additional file 4.5.2. Funnel plots of rs72552713 on gout in Asians.** A) OR_1_ in Asians B) OR_2_ in Asians


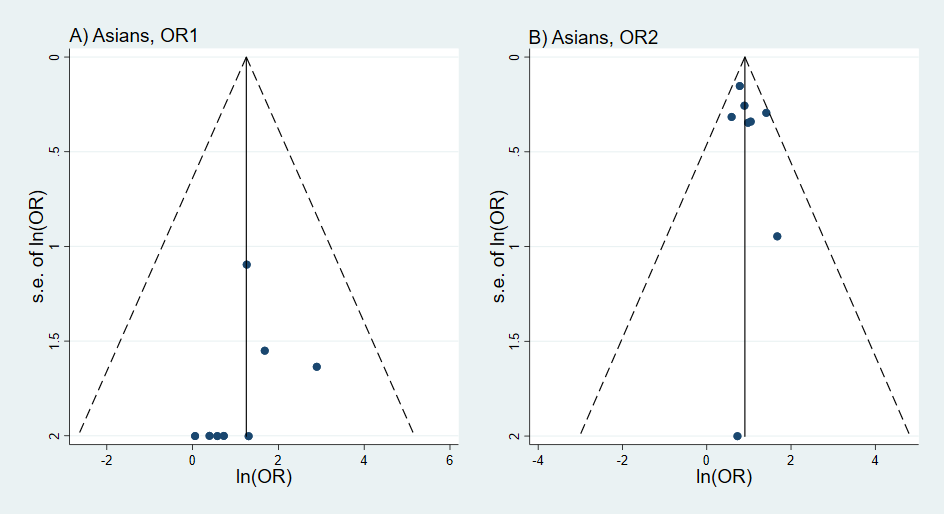


### **Additional file 4.5.3. Funnel plots of rs2231137 on gout in Asians.** A) OR_1_ in Asians B) OR_2_ in Asians

**
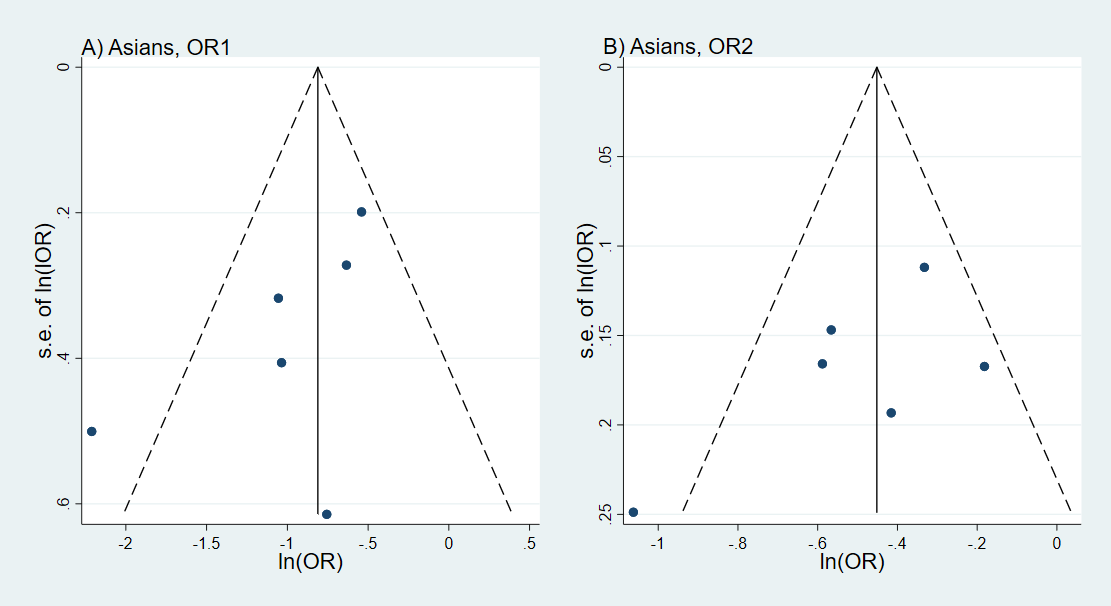
**

### **Additional file 4.5.4**. Funnel plots of rs1014290 on gout in Asians and Caucasians. A) OR_1_ in Asians B) OR_2_ in Asians C) OR_1_ in Caucasians D) OR_2_ in Caucasians

**
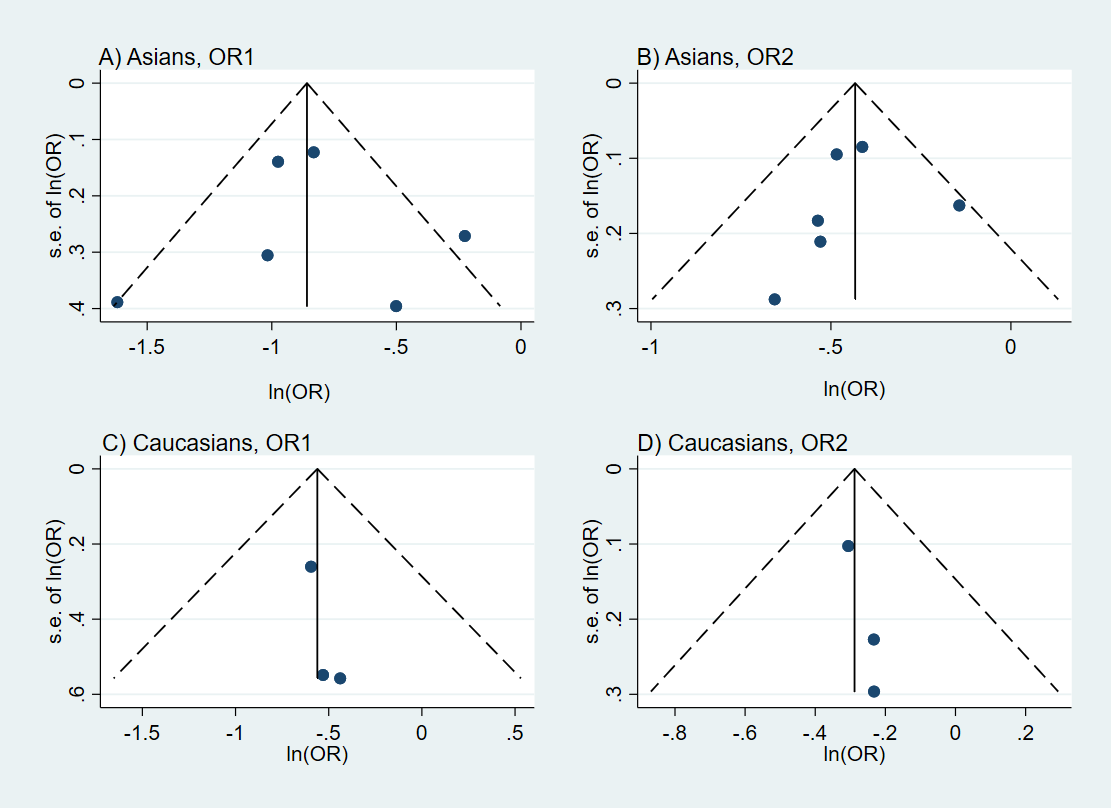
**

### **Additional file 4.5.5.** Funnel plots of rs3733591 on gout in Asians and Caucasians. A) OR_1_ in Asians B) OR_2_ in Asians C) OR_1_ in Caucasians D) OR_2_ in Caucasians


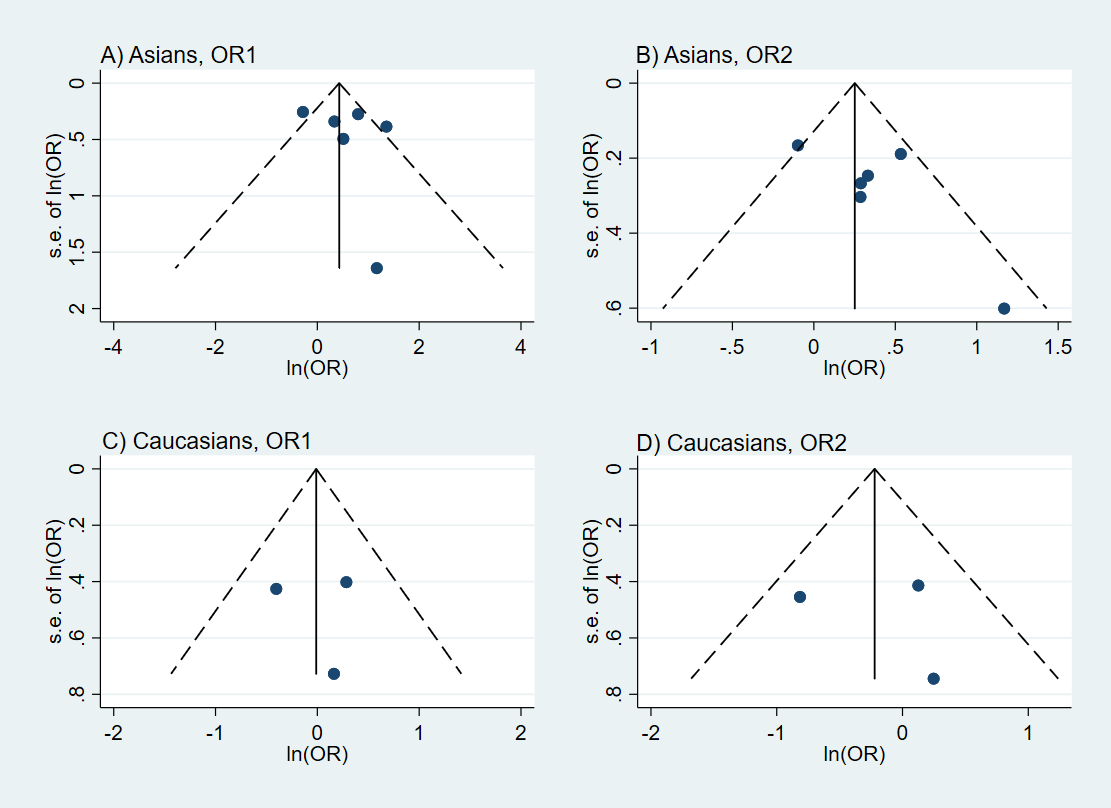


### **Additional file 4.5.6**. Funnel plots of rs6855911 on gout in Asians and Caucasians. A) OR_1_ in Asians B) OR_2_ in Asians C) OR_1_ in Caucasians D) OR_2_ in Caucasians


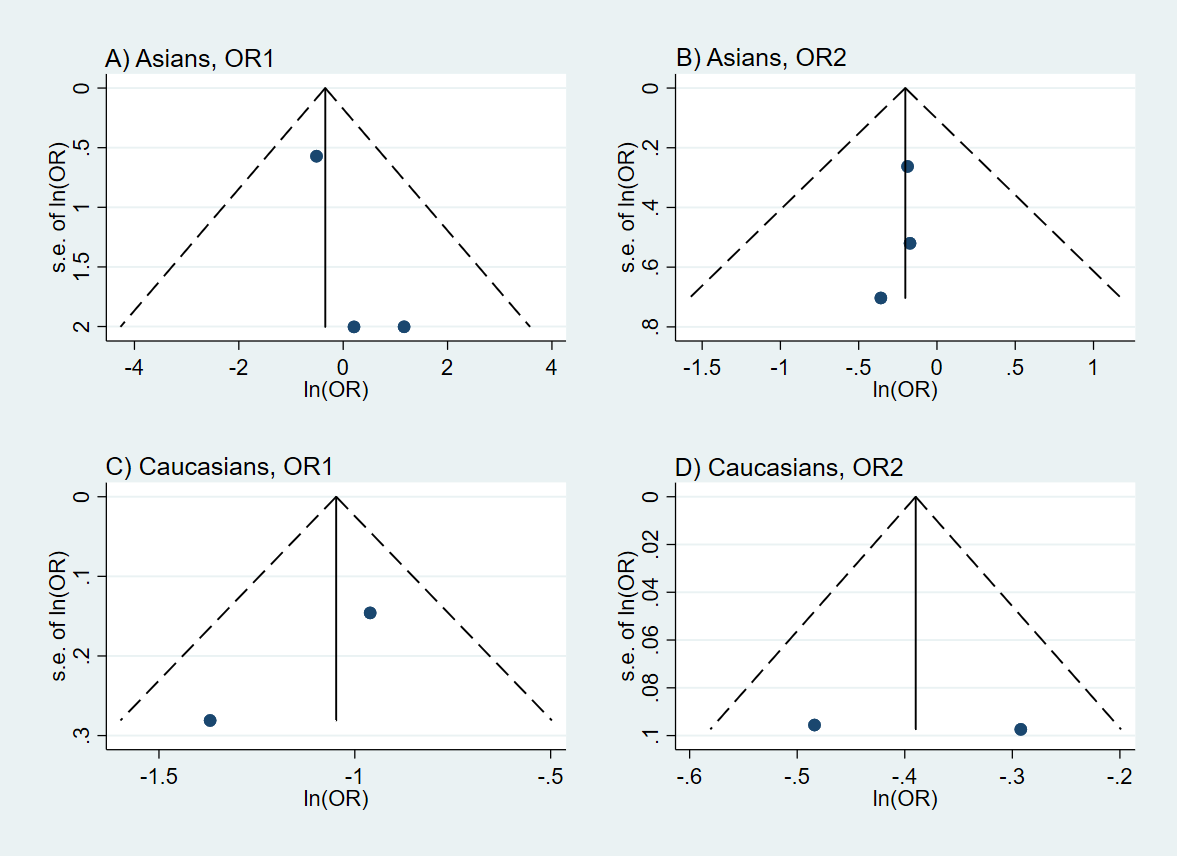


### Additional file 4.5.7. Funnel plots of rs16890979 on gout in Asians. A) OR_1_ in Asians B) OR_2_ in Asians

**
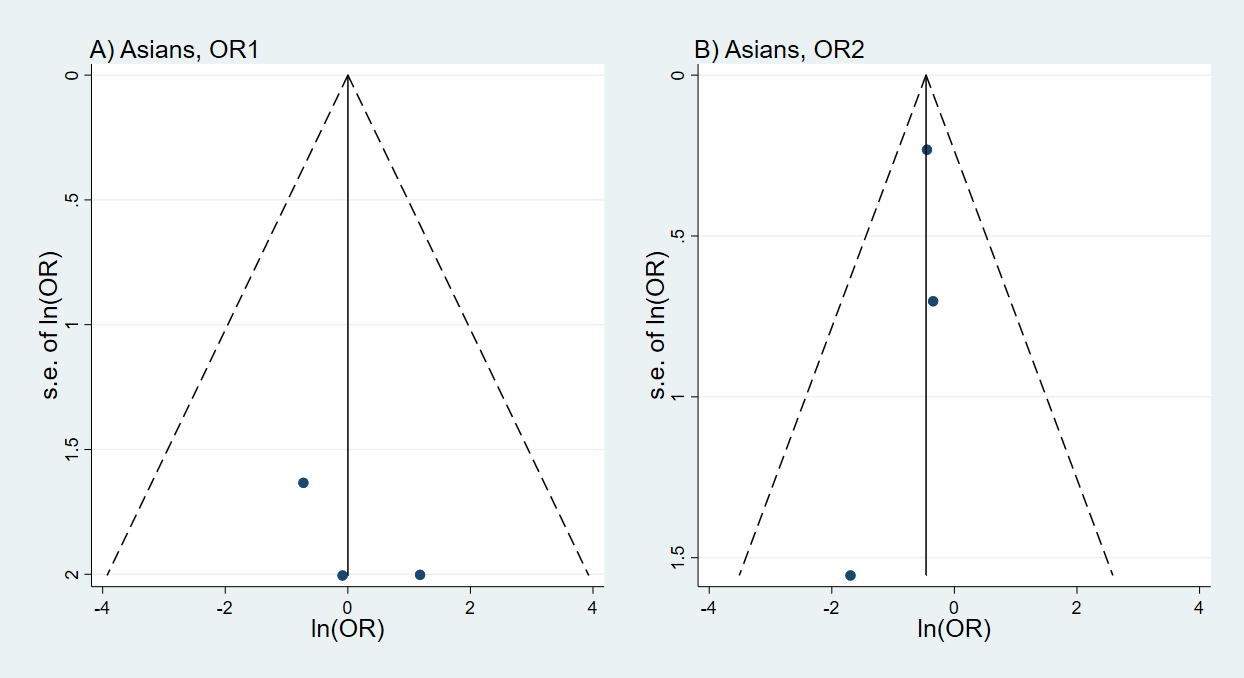
**

### **Additional file 4.5.8**. Funnel plots of rs6449213 on gout in Caucasians. A) OR_1_ in Caucasians B) OR_2_ in Caucasians


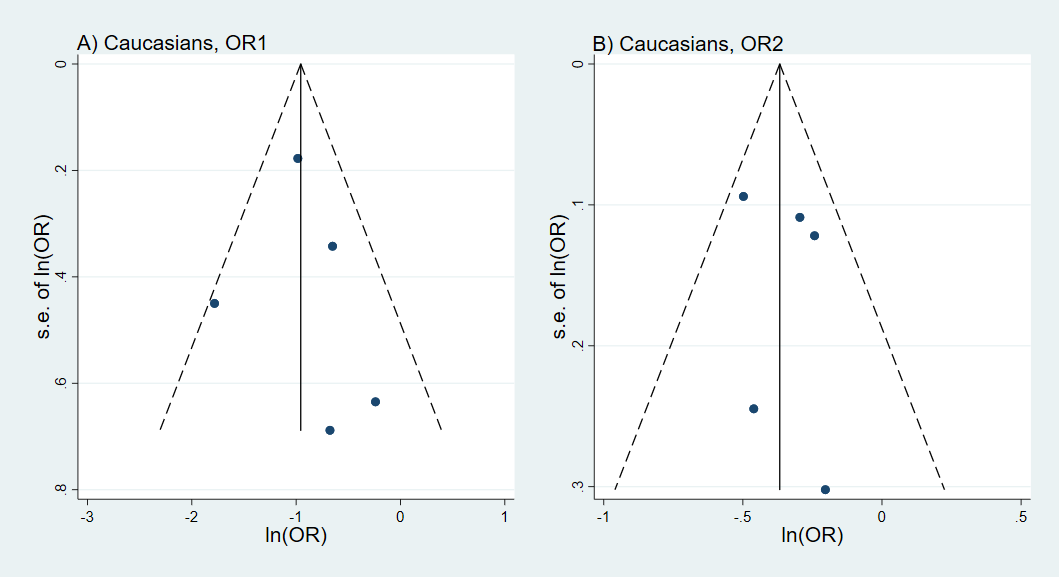


### **Additional file 4.5.9**. Funnel plots of rs7442295 on gout in Caucasians. A) OR_1_ in Caucasians B) OR_2_ in Caucasians

**
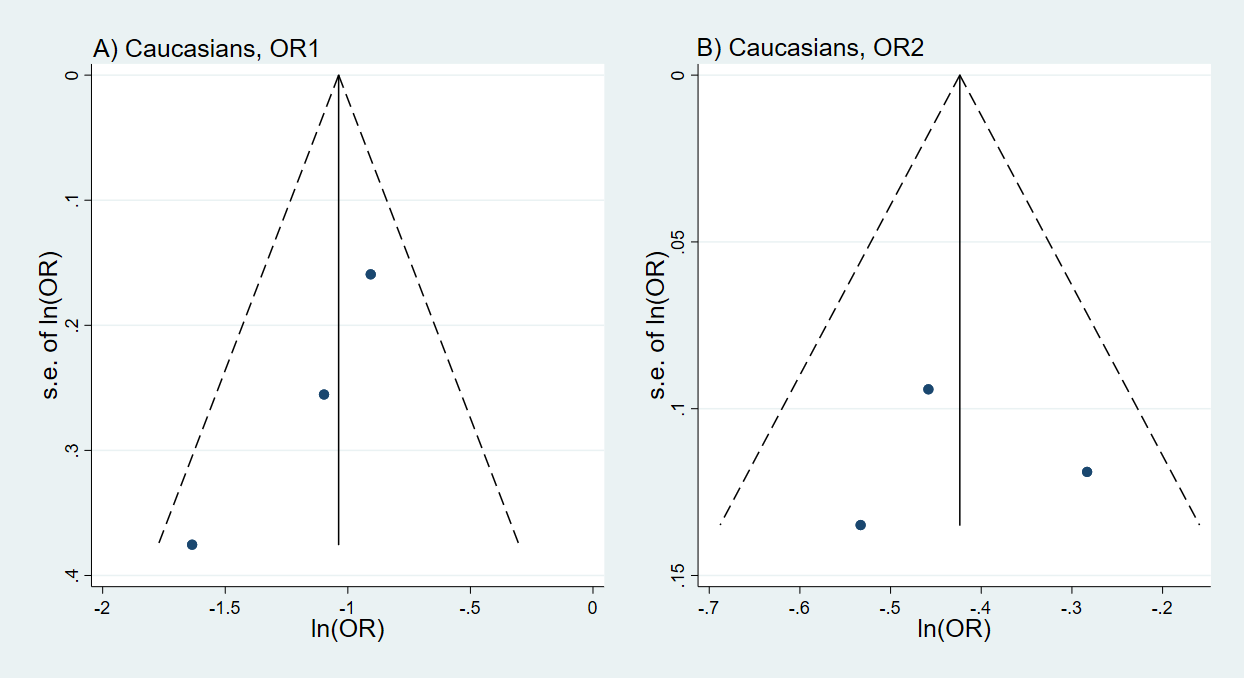
**

### **Additional file 4.5.10**. Funnel plots of rs12510549 on gout in Caucasians. A) OR_1_ in Caucasians B) OR_2_ in Caucasians

**
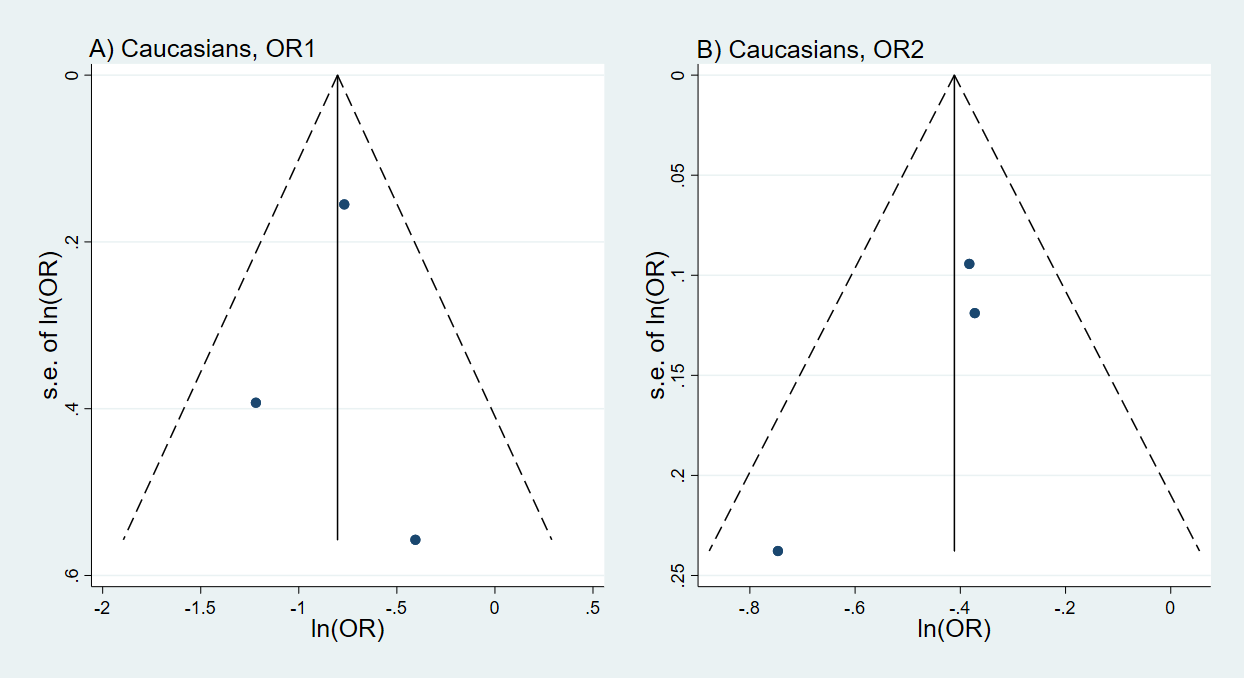
**
