# Supplementary material for: The association between genetic polymorphisms in ABCG2 and SLC2A9 and urate: an updated systematic review and meta-analysis
Source: BMC Med Genet. 2020 Oct 21;21:210. doi: 10.1186/s12881-020-01147-2 (PMC7580000; doi:10.1186/s12881-020-01147-2)

# Additional file 5. Hyperuricemia

## Additional file 5.1. Data used for pooling effects of *ABCG2*-rs2231142 on hyperuricemia

| Author | Year | Case | | | | Control | | | | HWE | AA/CC | | CA/CC | |
| --- | --- | --- | --- | --- | --- | --- | --- | --- | --- | --- | --- | --- | --- | --- |
|  |  | No. of subjects | Genotype | | | No. of subjects | Genotype | | | p-value | OR_1_ | 95% CI | OR_2_ | 95% CI |
|  |  |  | CC | CA | AA |  | CC | CA | AA |  |  |  |  |  |
| **Asians** | | | | | | | | | | | | | | |
| Matsuo H.^a^ | 2009 | 226 | 68 | 113 | 45 | 865 | 462 | 316 | 87 | 0.003 | 3.51 | 2.26, 5.46 | 2.43 | 1.74, 3.39 |
| Takeuchi F. | 2013 | 237 | 80 | 115 | 42 | 3218 | 1664 | 1300 | 254 | 0.997 | 3.44 | 2.31, 5.11 | 1.84 | 1.37, 2.47 |
| Tu HP. | 2016 | 106 | 48 | 43 | 15 | 295 | 147 | 123 | 25 | 0.919 | 1.84 | 0.90, 3.77 | 1.07 | 0.67, 1.72 |
| Chen CJ. | 2018 | 746 | 304 | 352 | 90 | 2070 | 1088 | 812 | 170 | 0.287 | 1.89 | 1.42, 2.52 | 1.55 | 1.30, 1.85 |
| Pooled OR | | | | | | | | | |  | 2.25 | 1.80, 2.81 | 1.56 | 1.35, 1.80 |
| **Mixed** | | | | | | | | | | | | | | |
| Kannangara DR. | 2016 | 448 | 312 | 111 | 25 | 344 | 287 | 53 | 4 | 0.324 | 5.75 | 1.98, 16.72 | 1.93 | 1.34, 2.77 |

^a^ Not included in pooling due to non-compliance with HWE.

*ABCG2*, ATP-binding cassette sub-family G member 2; CI, confidence interval; OR, odds ratio.

## Additional file 5.2. Exploring source of heterogeneity for *ABCG2*-rs2231142 on hyperuricemia

|  | No. of  sub-studies | OR_1_ | | | OR_2_ | | |
| --- | --- | --- | --- | --- | --- | --- | --- |
|  |  | OR | 95% CI | I^2^ (%) | OR | 95% CI | I^2^ (%) |
| ***ABCG2*** | | | | | | | |
| rs2231142 (Asians) | | | | | | | |
| Overall | 3 | 2.25 | 1.80, 2.81 | 67.2 | 1.56 | 1.35, 1.80 | 44.6 |
| Source of heterogeneity |  |  | |  |  | |  |
| Age | 3 | 2.25 | 1.80, 2.81 | 80.5 | 1.56 | 1.35, 1.80 | 66.8 |
| BMI | 3 | 2.25 | 1.80, 2.81 | 0.0 | 1.56 | 1.35, 1.80 | 55.7 |
| Percent male^a^ | 3 | 2.25 | 1.80, 2.81 | 0.0 | 1.56 | 1.35, 1.80 | 51.1 |
| Subgroup analysis |  |  | |  |  | |  |
| %male < 100 | 1 | 3.44 | 2.31, 5.11 | NA | 1.84 | 1.37, 2.47 | NA |
| %male = 100 | 2 | 1.89 | 0.90, 3.77 | 0.0 | 1.38 | 0.98, 1.93 | 51.1 |

^a^ Categorized variable

*ABCG2*, ATP-binding cassette sub-family G member 2; BMI, body mass index; CI, confidence interval; NA, not applicable; OR, odds ratio.

## Additional file 5.3. Egger’s tests for *ABCG2*-rs2231142 on hyperuricemia

|  | No. of sub-studies | OR_1_ | | | OR_2_ | | |
| --- | --- | --- | --- | --- | --- | --- | --- |
|  |  | Coef. of Egger’s test | SE | *P* value | Coef. of Egger’s test | SE | *P* value |
| ***ABCG2*** |  |  |  |  |  |  |  |
| rs2231142 |  |  |  |  |  |  |  |
| - Asians | 3 | 0.69 | 4.27 | 0.898 | -1.37 | 2.68 | 0.700 |

*ABCG2*, ATP-binding cassette sub-family G member 2; Coef., coefficient; OR, odds ratio; SE, standard error.

## Additional file 5.4. Funnel plots of *ABCG2*-rs2231142 on hyperuricemia in Asians. A) OR_1_ in Asians B) OR_2_ in Asians


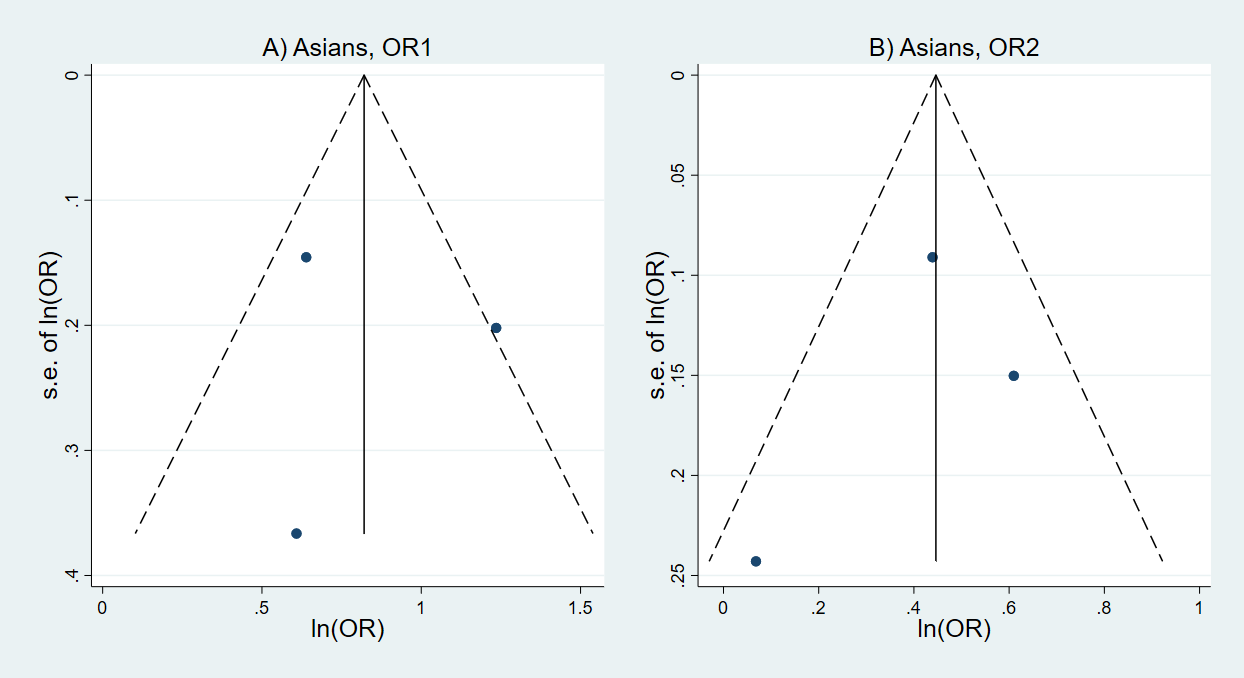

Supplement: Supplementary file 5 — Hyperuricemia. 5.1. Data used for pooling effects of ABCG2-rs2231142 on hyperuricemia. 5.2. Exploring source of heterogeneity for ABCG2-rs2231142 on hyperuricemia. 5.3. Egger’s tests for ABCG2-rs2231142 on hyperuricemia. 5.4. Funnel plots of ABCG2-rs2231142 on hyperuricemia in Asians. A) OR1 in Asians B) OR2 in Asians. (DOCX 77 kb) [file 12881_2020_1147_MOESM5_ESM.docx]
