# Supplementary material for: The association between genetic polymorphisms in ABCG2 and SLC2A9 and urate: an updated systematic review and meta-analysis
Source: BMC Med Genet. 2020 Oct 21;21:210. doi: 10.1186/s12881-020-01147-2 (PMC7580000; doi:10.1186/s12881-020-01147-2)

# Additional file 6. Serum urate

## Additional file 6.1. Data used for pooling mean difference of *ABCG2* and *SLC2A9* polymorphisms on serum urate

| *ABCG2* rs2231142 | | | | | | | | | | | | | | | |
| --- | --- | --- | --- | --- | --- | --- | --- | --- | --- | --- | --- | --- | --- | --- | --- |
| Author | Year | Genotype | | | | | | | | | HWE | AA/CC | | CA/CC | |
|  |  | CC | | | CA | | | AA | | | p-value | MD_1_ (µmol/l) | 95% CI | MD_2_ (µmol/l) | 95% CI |
|  |  | No. of subjects | Mean urate (µmol/l) | SD | No. of subjects | Mean urate (µmol/l) | SD | No. of subjects | Mean urate (µmol/l) | SD |  |  |  |  |  |
| **Caucasians** | | | | | | | | | | | | | | | |
| Brandstatter A. | 2010 |  |  |  |  |  |  |  |  |  |  |  |  |  |  |
| - Bruneck |  | 612 | 270.63 | 73.57 | 178 | 294.43 | 71.42 | 10 | 305.13 | 67.71 | 0.463 | 34.50 | 21.22, 47.77 | 23.79 | 11.79, 35.79 |
| - SAPHIR |  | 1339 | 328.33 | 65.30 | 358 | 347.36 | 67.52 | 35 | 371.75 | 70.38 | 0.058 | 43.42 | 39.48, 47.36 | 19.03 | 11.21, 26.85 |
| Stiburkova B. | 2014 | 475 | 271.52 | 43.62 | 108 | 291.56 | 47.90 | 6 | 373 | 49.56 | 0.960 | 101.48 | 61.63, 141.33 | 20.04 | 10.19, 29.89 |
| Bartakova V. | 2016 | 344 | 365.33 | 88.59 | 74 | 372.00 | 79.39 | 4 | 357.00 | 75.35 | 0.993 | -8.33 | -82.77, 66.10 | 6.67 | -13.70, 27.03 |
| Pooled OR |  |  |  |  |  |  |  |  |  |  |  | 50.43 | 32.66, 68.20 | 19.46 | 14.19, 24.73 |
| **Asians** | | | | | | | | | | | | | | | |
| Matsuo H. | 2009 | 374 | 287.88 | 72.57 | 297 | 299.78 | 77.32 | 68 | 333.68 | 85.06 | 0.417 | 45.80 | 24.29, 67.31 | 11.90 | 0.43, 23.36 |
| Tabara Y. | 2010 |  |  |  |  |  |  |  |  |  |  |  |  |  |  |
| - Ehime |  | 1590 | 300.60 | 73.74 | 951 | 311.98 | 73.63 | 153 | 322.94 | 73.84 | 0.492 | 22.34 | 10.09, 34.59 | 11.38 | 5.46, 17.30 |
| - Suita |  | 1254 | 303.92 | 68.43 | 1018 | 308.55 | 71.05 | 199 | 314.20 | 81.76 | 0.704 | 10.28 | -1.69, 22.25 | 4.63 | -1.15, 10.41 |
| Yamagishi K. | 2010 | 1861 | 276.85 | 66.05 | 1673 | 286.64 | 66.77 | 389 | 288.07 | 70.97 | 0.647 | 11.22 | 3.56, 18.89 | 9.79 | 5.41, 14.18 |
| Hu M.^a^ | 2012 | 172 | 359.56 | 87.90 | 129 | 350.27 | 83.70 | 48 | 339.67 | 93.00 | 0.004 | -19.90 | -49.30, 9.51 | -9.29 | -28.82, 10.23 |
| Cheng ST. | 2017 | 208 | 358.07 | 92.79 | 202 | 391.38 | 96.36 | 41 | 378.89 | 98.14 | 0.418 | 20.82 | -11.76, 53.40 | 33.31 | 14.99, 51.63 |
| Pooled OR |  |  |  |  |  |  |  |  |  |  |  | 18.41 | 8.68, 28.14 | 11.49 | 4.10, 18.89 |
| *SLC2A9* rs1014290 | | | | | | | | | | | | | | | |
| Author | Year | Genotype | | | | | | | | | HWE | CC/TT | | TC/TT | |
|  |  | TT | | | TC | | | CC | | | p-value | MD_1_ (µmol/l) | 95% CI | MD_2_ (µmol/l) | 95% CI |
|  |  | No. of subjects | Mean urate (µmol/l) | SD | No. of subjects | Mean urate (µmol/l) | SD | No. of subjects | Mean urate (µmol/l) | SD |  |  |  |  |  |
| **Caucasians** | | | | | | | | | | | | | | | |
| Vitart V. | 2008 |  |  |  |  |  |  |  |  |  |  |  |  |  |  |
| - Croatia |  | 375 | 304.47 | 30.14 | 332 | 290.39 | 27.35 | 87 | 260.50 | 15.5 | 0.297 | -43.97 | -48.43, -39.51 | -14.08 | -18.32, -9.84 |
| - Scotland   (Orkney) |  | 391 | 310.45 | 25.39 | 220 | 293.00 | 18.93 | 38 | 273.82 | 7.52 | 0.343 | -36.64 | -40.11, -33.16 | -17.45 | -21.00, -13.90 |
| Bartakova V. | 2016 | 230 | 363.33 | 83.55 | 163 | 370.17 | 87.51 | 29 | 389.67 | 115.40 | 0.987 | 26.33 | -17.03, 69.70 | 6.83 | -10.40, 24.07 |
| Pooled OR |  |  |  |  |  |  |  |  |  |  |  | -32.33 | -52.61, -12.05 | -9.87 | -25.27, 5.54 |
| **Asians** | | | | | | | | | | | | | | | |
| Tabara Y. | 2010 |  |  |  |  |  |  |  |  |  |  |  |  |  |  |
| - Ehime |  | 761 | 315.99 | 73.76 | 1330 | 305.70 | 73.76 | 603 | 291.48 | 70.78 | 0.633 | -24.51 | -32.22, -16.81 | -10.30 | -16.87, -3.73 |
| - Suita |  | 819 | 314.02 | 70.78 | 1214 | 306.42 | 68.40 | 438 | 291.57 | 89.22 | 0.746 | -22.44 | -32.10, -12.78 | -7.60 | -13.79, -1.41 |
| Urano W. | 2010 | 204 | 333.68 | 63.05 | 279 | 342.60 | 69.00 | 94 | 334.87 | 77.92 | 0.932 | 1.19 | -16.78, 19.16 | 8.92 | -2.93, 20.77 |
| Liu WC. | 2011 | 324 | 361.67 | 76.70 | 513 | 354.45 | 96.19 | 166 | 345.50 | 91.73 | 0.121 | -16.17 | -32.44, 0.09 | -7.22 | -19.01, 4.57 |
| Hu M. | 2012 | 144 | 376.93 | 81.5 | 151 | 344.97 | 83.5 | 51 | 313.39 | 92.8 | 0.271 | -63.54 | -92.28, -34.80 | -31.96 | -50.79, -13.13 |
| Zhang XY. | 2015 | 139 | 409.07 | 141.52 | 143 | 398.84 | 155.20 | 30 | 401.52 | 145.36 | 0.436 | -7.55 | -64.64, 49.54 | -10.23 | -44.88, 24.42 |
| Pooled OR |  |  |  |  |  |  |  |  |  |  |  | -21.47 | -30.97, -11.96 | -8.16 | -16.63, 0.31 |
| *SLC2A9* rs6449213 | | | | | | | | | | | | | | | |
| Author | Year | Genotype | | | | | | | | | HWE | CC/TT | | TC/TT | |
|  |  | TT | | | TC | | | CC | | | p-value | MD_1_ (µmol/l) | 95% CI | MD_2_ (µmol/l) | 95% CI |
|  |  | No. of subjects | Mean urate (µmol/l) | SD | No. of subjects | Mean urate (µmol/l) | SD | No. of subjects | Mean urate (µmol/l) | SD |  |  |  |  |  |
| **Caucasians** | | | | | | | | | | | | | | | |
| Brandstatter A. | 2008 |  |  |  |  |  |  |  |  |  |  |  |  |  |  |
| - Utah |  | 539 | 330.11 | 91.14 | 259 | 297.40 | 92.85 | 20 | 271.82 | 112.25 | 0.087 | -58.29 | -108.08, -8.50 | -32.71 | -46.39, -19.04 |
| Vitart V. | 2008 |  |  |  |  |  |  |  |  |  |  |  |  |  |  |
| - Croatia |  | 456 | 303.18 | 46.12 | 277 | 286.57 | 36.95 | 56 | 250.09 | 15.49 | 0.123 | -53.09 | -58.96, -47.23 | -16.61 | -22.68, -10.54 |
| - Scotland (Orkney) |  | 429 | 308.43 | 37.90 | 190 | 292.75 | 24.54 | 22 | 278.31 | 8.91 | 0.865 | -30.12 | -35.29, -24.95 | -15.68 | -20.69, -10.68 |
| Brandstatter A. | 2010 |  |  |  |  |  |  |  |  |  |  |  |  |  |  |
| - Bruneck |  | 525 | 286.10 | 68.13 | 245 | 262.31 | 65.14 | 31 | 228.40 | 66.45 | 0718 | -57.70 | -81.80, -33.59 | -23.79 | -33.82, -13.77 |
| - SAPHIR |  | 1114 | 341.42 | 79.40 | 542 | 322.98 | 69.24 | 76 | 293.24 | 67.50 | 0.331 | -48.18 | -64.05, -32.30 | -18.44 | -25.90, -10.97 |
| Voruganti VS. | 2013 | 384 | 327.14 | 83.27 | 217 | 309.30 | 83.27 | 31 | 249.82 | 71.38 | 0.962 | -77.32 | -103.79, -50.85 | -17.84 | -31.70, -3.98 |
| Pooled OR |  |  |  |  |  |  |  |  |  |  |  | -49.29 | -59.85, -38.74 | -20.43 | -28.91, -11.94 |
| **Asians** | | | | | | | | | | | | | | | |
| Urano W. | 2010 | 566 | 338.44 | 68.40 | 9 | 344.39 | 49.37 | 1 | 368.78 | 0 | 0.047 | NA | NA | 5.95 | -26.79, 38.69 |
| **American Indians** | | | | | | | | | | | | | | | |
| Laston SL. | 2015 | 459 | 365.21 | 95.17 | 432 | 344.98 | 95.17 | 102 | 309.89 | 89.22 | 0.981 | -55.32 | -74.70, -35.94 | -20.22 | -32.73, -7.72 |
| **Mixed** | | | | | | | | | | | | | | | |
| Cummings N. | 2010 | 260 | 277.34 | 78.79 | 110 | 259.91 | 86.32 | 12 | 193.25 | 82.18 | 0.930 | -84.09 | -131.56, -36.62 | -17.43 | -36.19, 1.33 |
| *SLC2A9* rs6855911 | | | | | | | | | | | | | | | |
| Author | Year | Genotype | | | | | | | | | HWE | GG/AA | | AG/AA | |
|  |  | AA | | | AG | | | GG | | | p-value | MD_1_ (µmol/l) | 95% CI | MD_2_ (µmol/l) | 95% CI |
|  |  | No. of subjects | Mean urate (µmol/l) | SD | No. of subjects | Mean urate (µmol/l) | SD | No. of subjects | Mean urate (µmol/l) | SD |  |  |  |  |  |
| **Caucasians** | | | | | | | | | | | | | | | |
| Li S. | 2007 |  |  |  |  |  |  |  |  |  |  |  |  |  |  |
| - InCHIANTI |  | 706 | 313.46 | 85.65 | 485 | 293.83 | 77.92 | 85 | 257.55 | 81.49 | 0.890 | -55.91 | -74.35, -37.47 | -19.63 | -29.01, -10.25 |
| - SardiNIA |  | 715 | 277.18 | 89.81 | 508 | 266.47 | 94.57 | 87 | 239.11 | 96.95 | 0.800 | -38.07 | -59.48, -16.66 | -10.71 | -21.24, -0.17 |
| Brandstatter A. | 2008 |  |  |  |  |  |  |  |  |  |  |  |  |  |  |
| - Utah |  | 471 | 330.71 | 92.94 | 304 | 303.94 | 92.30 | 43 | 288.48 | 98.68 | 0.502 | -42.23 | -72.90 -11.57 | -26.77 | -40.11, -13.42 |
| Brandstatter A. | 2010 |  |  |  |  |  |  |  |  |  |  |  |  |  |  |
| - Bruneck |  | 467 | 287.88 | 64.23 | 290 | 265.88 | 70.95 | 43 | 232.57 | 66.46 | 0.817 | -55.32 | -76.02 -34.62 | -22.01 | -32.04, -11.98 |
| - SAPHIR |  | 935 | 344.98 | 72.76 | 672 | 324.76 | 77.10 | 125 | 289.07 | 66.42 | 0.778 | -55.91 | -68.45 -43.37 | -20.22 | -27.69, -12.76 |
| Lyngdoh T. | 2012 | 2684 | 325.5 | 82.3 | 2142 | 303.5 | 83.6 | 398 | 275.4 | 83.4 | 0.302 | -50.10 | -58.87 -41.33 | -22.00 | -26.71, -17.29 |
| Pooled OR |  |  |  |  |  |  |  |  |  |  |  | -51.23 | -57.22, -45.25 | -20.65 | -23.83, -17.47 |
| **Asians** | | | | | | | | | | | | | | | |
| Urano W. | 2010 | 570 | 339.04 | 68.402 | 11 | 337.85 | 90.41 | 0 | NA | NA | 1.000 | NA | NA | -1.19 | -54.91, 52.53 |
| Guan M. | 2011 | 357 | 380.7 | 130.8 | 15 | 284.5 | 97.6 | 0 | NA | NA | 1.000 | NA | NA | -96.20 | -147.42, -44.98 |
| **Mixed** | | | | | | | | | | | | | | | |
| Cummings N. | 2010 | 196 | 277.68 | 78.23 | 160 | 265.71 | 84.02 | 32 | 230.67 | 67.34 | 0.935 | -47.01 | -72.78, -21.24 | -11.97 | -28.98, 5.04 |
| *SLC2A9* rs7442295 | | | | | | | | | | | | | | | |
| Author | Year | Genotype | | | | | | | | | HWE | GG/AA | | AG/AA | |
|  |  | AA | | | AG | | | GG | | | p-value | MD_1_ (µmol/l) | 95% CI | MD_2_ (µmol/l) | 95% CI |
|  |  | No. of subjects | Mean urate (µmol/l) | SD | No. of subjects | Mean urate (µmol/l) | SD | No. of subjects | Mean urate (µmol/l) | SD |  |  |  |  |  |
| **Caucasians** | | | | | | | | | | | | | | | |
| Li S. | 2007 |  |  |  |  |  |  |  |  |  |  |  |  |  |  |
| - InCHIANTI |  | 764 | 312.27 | 85.06 | 443 | 293.83 | 79.70 | 68 | 245.65 | 71.38 | 0.718 | -66.62 | -84.62, -48.61 | -18.44 | -28.00, -8.88 |
| - SardiNIA |  | 882 | 279.56 | 90.41 | 455 | 264.09 | 95.76 | 55 | 228.40 | 79.70 | 0.699 | -51.15 | -73.05, -29.26 | -15.46 | -26.10, -4.83 |
| Brandstatter A. | 2008 |  |  |  |  |  |  |  |  |  |  |  |  |  |  |
| - Utah |  | 501 | 330.71 | 91.86 | 287 | 299.78 | 92.70 | 28 | 286.69 | 108.90 | 0.090 | -44.02 | -85.15, -2.88 | -30.93 | -44.34, -17.52 |
| Brandstatter A. | 2010 |  |  |  |  |  |  |  |  |  |  |  |  |  |  |
| - Bruneck |  | 500 | 286.69 | 66.50 | 265 | 264.09 | 67.75 | 34 | 231.97 | 66.28 | 0.881 | -54.72 | -77.75, -31.69 | -22.60 | -32.63, -12.58 |
| - SAPHIR |  | 1037 | 343.20 | 76.63 | 601 | 322.38 | 72.91 | 94 | 290.86 | 69.03 | 0.571 | -52.34 | -67.06, -37.63 | -20.82 | -28.28, -13.35 |
| Kobylecki CJ. | 2017 | 53769 | 303.33 | 81.55 | 28739 | 286.67 | 88.96 | 3702 | 250 | 88.99 | 0.077 | -53.33 | -56.28, -50.38 | -16.67 | -17.90, -15.43 |
| Pooled OR |  |  |  |  |  |  |  |  |  |  |  | -54.30 | -58.78, -49.82 | -18.34 | -22.68, -13.99 |
| **Asians** | | | | | | | | | | | | | | | |
| Urano W. | 2010 | 578 | 339.04 | 67.81 | 9 | 339.63 | 93.98 | 0 | NA | NA | 1.000 | NA | NA | 0.59 | -61.05, 62.24 |
| **Mixed** | | | | | | | | | | | | | | | |
| Cummings N. | 2010 | 186 | 226.38 | 66.09 | 160 | 270.53 | 83.55 | 34 | 276.94 | 78.14 | 0.961 | 50.56 | 22.63, 78.49 | 44.15 | 28.09, 60.21 |
| *SLC2A9* rs734553 | | | | | | | | | | | | | | | |
| Author | Year | Genotype | | | | | | | | | HWE | GG/TT | | TG/TT | |
|  |  | TT | | | TG | | | GG | | | p-value | MD_1_ (µmol/l) | 95% CI | MD_2_ (µmol/l) | 95% CI |
|  |  | No. of subjects | Mean urate (µmol/l) | SD | No. of subjects | Mean urate (µmol/l) | SD | No. of subjects | Mean urate (µmol/l) | SD |  |  |  |  |  |
| **Caucasians** | | | | | | | | | | | | | | | |
| Testa A. | 2014 | 97 | 292.64 | 83.27 | 97 | 265.2808 | 79.70 | 17 | 243.87 | 88.63 | 0.284 | -48.77 | -94.04, -3.50 | -27.36 | -50.30, -4.42 |
| Mallamaci F. | 2015 | 217 | 309.30 | 83.27 | 198 | 279.556 | 77.32 | 34 | 249.82 | 95.17 | 0.222 | -59.48 | -93.33, -25.63 | -29.74 | -45.19, -14.29 |
| Bartakova V. | 2016 | 244 | 367.67 | 85.02 | 154 | 361.67 | 90.55 | 24 | 402 | 117.42 | 0.963 | 34.33 | -13.84, 82.51 | -6.00 | -23.84, 11.84 |
| Pooled OR |  |  |  |  |  |  |  |  |  |  |  | -29.29 | -63.90, 5.33 | -20.68 | -47.08, 5.71 |
| **American Indians** | | | | | | | | | | | | | | | |
| Laston SL. | 2015 | 258 | 330.71 | 95.17 | 496 | 349.74 | 95.17 | 239 | 371.16 | 95.17 | 0.984 | 40.45 | 23.70, 57.19 | 19.03 | 4.72, 33.35 |

^a^ Not included in pooling due to non-compliance with HWE.

*ABCG2*, ATP-binding cassette sub-family G member 2; CI, confidence interval; InCHIANTI, Invecchiare in Chianti, aging in the Chianti area; MD, mean difference; SAPHIR, Salzburg Atherosclerosis Prevention Program in Subjects at High Individual Risk; SD, standard deviation; *SLC2A9*, solute carrier family 2 member 9.

## Additional file 6.2. Exploring source of heterogeneity for *ABCG2* and *SLC2A9* polymorphisms on serum urate

|  | No. of  sub-studies | MD_1_ | | | MD_2_ | | | |
| --- | --- | --- | --- | --- | --- | --- | --- | --- |
|  |  | MD | 95% CI | I^2^ (%) | MD | 95% CI | I^2^ (%) | |
| ***ABCG2*** | | | | | | | | |
| rs2231142 (Caucasians) | | | | | | | | |
| Overall | 4 | 50.43 | 32.66, 68.20 | 68.7 | 19.46 | 14.19, 24.73 | 0.0 | |
| Source of heterogeneity |  |  |  |  |  | |  | |
| Age | 3 |  |  | 79.1 |  |  | NA | |
| BMI | 3 |  |  | 83.7 |  |  | NA | |
| Percent male^a^ | 4 |  |  | 70.5 |  |  | NA | |
| Type of population^a^ | 4 |  |  | 71.6 |  |  | NA | |
| rs2231142 (Asians) | | | | | | | | |
| Overall | 5 | 18.41 | 8.68, 28.14 | 63.1 | 11.49 | 4.10, 18.89 | 59.1 | |
| Source of heterogeneity |  |  |  |  |  |  |  | |
| Age^a^ | 5 |  |  | 72.1 |  |  | 5.9 | |
| BMI | 4 |  |  | 47.6 |  |  | 66.2 | |
| Percent male^a^ | 5 |  |  | 72.1 |  |  | 5.9 | |
| Subgroup analysis | 5 |  |  |  |  |  |  | |
| Mean age < 60 & %male ≥ 50 | 1 | 20.82 | -6.65, 48.29 | NA | 33.31 | 14.99, 51.63 | NA | |
| Mean age ≥ 60 & %male < 50 | 4 | 16.22 | 9.09, 23.36 | 72.1 | 9.12 | 4.85, 13.39 | 5.9 | |
| ***SLC2A9*** | | | | | | | | |
| rs1014290 (Caucasians) | | | | | | | | |
| Overall | 3 | -32.33 | -52.61,-12.05 | 86.9 | -9.87 | -25.27,5.54 | 75.1 | |
| Source of heterogeneity |  |  |  |  |  |  |  | |
| Percent male^a^ | 3 |  |  | 84.5 |  |  | 30.0 | |
| Type of population^a^ | 3 |  |  | 84.5 |  |  | 30.0 | |
| Subgroup analysis |  |  |  |  |  |  |  | |
| General population & %male < 50 | 2 | -40.16 | -47.74, -32.57 | 84.5 | -15.80 | -23.36, -8.24 | 30.0 | |
| Specific population (T2D) & %male ≥ 50 | 1 | 26.33 | -17.03, 69.70 | NA | 6.83 | -10.40, 24.07 | NA | |
| rs1014290 (Asians) | | | | | | | | |
| Overall | 6 | -21.47 | -30.97, -11.96 | 67.9 | -8.16 | -16.63, 0.31 | 65.7 | |
| Source of heterogeneity |  |  |  |  |  |  |  | |
| Age | 4 |  |  | NA^b^ |  |  | NA^b^ | |
| BMI | 3 |  |  | NA^c^ |  |  | NA^c^ | |
| Percent male^a^ | 6 |  |  | 53.5 |  |  | 33.4 | |
| Type of population^a^ | 6 |  |  | 60.0 |  |  | 55.0 | |
| Subgroup analysis |  |  |  |  |  |  |  | |
| %male < 100 | 5 | -24.14 | -29.59, -18.70 | 53.5 | -9.78 | -13.85, -5.70 | 33.4 | |
| %male = 100 | 1 | 1.19 | -17.64, 20.02 | NA | 8.92 | -4.06, 21.90 | NA | |
| rs6449213 (Caucasians) | | | | | | | | |
| Overall | 6 | -49.29 | -59.85, -38.74 | 88.5 | -20.43 | -28.91, -11.94 | 26.0 | |
| Source of heterogeneity |  |  |  |  |  |  |  | |
| Age | 6 |  |  | NA^b^ |  |  | NA^b^ | |
| BMI | 3 |  |  | NA^c^ |  |  | 68.4 | |
| %male^a^ | 5 |  |  | 91.6 |  |  | 55.6 | |
| rs7442295 (Caucasians) | | | | | | | | |
| Overall | 6 | -54.30 | -58.78, -49.82 | 0.0 | -18.34 | -22.68, -13.99 | | 26.4 |
| Source of heterogeneity |  |  |  |  |  |  | |  |
| Age^a^ | 4 |  |  | NA |  |  | | 33.2 |
| BMI | 4 |  |  | NA |  |  | | 35.4 |
| Percent male^a^ | 6 |  |  | NA |  |  | | 0.0 |
| Subgroup analysis |  |  |  |  |  |  | |  |
| %male ≥ 48 | 3 |  |  | NA | -23.03 | -28.50, -17.56 | | 0.0 |
| %male < 48 | 3 |  |  | NA | -16.67 | -17.89, -15.45 | | 0.0 |
| rs734553 (Caucasians) | | | | | | | | |
| Overall | 3 | -29.29 | -63.90, 5.33 | 80.6 | -20.68 | -47.08, 5.71 | | 52.8 |
| Source of heterogeneity |  |  |  |  |  |  | |  |
| Percent male^a^ | 3 |  |  | 0.0 |  |  | | 0.0 |
| Type of population^a^ | 3 |  |  | 0.0 |  |  | | 0.0 |
| Subgroup analysis |  |  |  |  |  |  | |  |
| General population & %male < 50 | 2 | -55.67 | -82.77, -28.57 | 0.0 | -29.00 | -41.82, -16.19 | | 0.0 |
| Specific population (T2D) & %male ≥ 50 | 1 | 34.33 | -13.84, 82.51 | NA | -6.00 | -23.84, 11.84 | | NA |

^a^ Categorized variables, ^b^ No heterogeneity among studies with data of age available, ^c^ No heterogeneity among studies with data of BMI available.

*ABCG2*, ATP-binding cassette sub-family G member 2; BMI, body mass index; CI, confidence interval; NA, not applicable; MD, mean difference; *SLC2A9*, solute carrier family 2 member 9, T2D, type 2 diabetes.

## Additional file 6.3. Egger’s tests for *ABCG2* and *SLC2A9* polymorphisms on serum urate

|  | No. of sub-studies | MD_1_ | | | MD_2_ | | |
| --- | --- | --- | --- | --- | --- | --- | --- |
|  |  | Coef. of Egger’s test | SE | *P* value | Coef. of Egger’s test | SE | *P* value |
| ***ABCG2*** |  |  |  |  |  |  |  |
| rs2231142 |  |  |  |  |  |  |  |
| - Caucasians | 4 | -0.66 | 2.92 | 0.843 | -1.32 | 1.39 | 0.443 |
| - Asians | 5 | 2.35 | 1.50 | 0.215 | 2.35 | 1.47 | 0.207 |
| **SLC2A9** |  |  |  |  |  |  |  |
| rs1014290 |  |  |  |  |  |  |  |
| - Caucasians | 3 | 2.62 | 3.30 | 0.572 | 3.51 | 0.99 | 0.174 |
| - Asians | 6 | 0.098 | 1.62 | 0.955 | -0.38 | 1.69 | 0.833 |
| rs6449213 |  |  |  |  |  |  |  |
| - Caucasians | 6 | -2.08 | 1.93 | 0.344 | -2.36 | 0.91 | 0.060 |
| rs6855911 |  |  |  |  |  |  |  |
| - Caucasians | 6 | 0.45 | 0.83 | 0.620 | 0.62 | 1.23 | 0.643 |
| rs7442295 |  |  |  |  |  |  |  |
| - Caucasians | 6 | -0.16 | 0.41 | 0.718 | -1.03 | 0.46 | 0.088 |
| rs734553 |  |  |  |  |  |  |  |
| - Caucasians | 3 | 8.87 | 7.67 | 0.454 | 0.69 | 7.57 | 0.942 |

*ABCG2*, ATP-binding cassette sub-family G member 2; Coef., coefficient; MD, mean difference; SE, standard error; *SLC2A9*, solute carrier family 2 member 9.

## Additional file 6.4. Funnel plots for *ABCG2* and *SLC2A9* polymorphisms on serum urate

### Additional file 6.4.1. Funnel plots of rs2231142 on serum urate in Asians and Caucasians. A) MD_1_ in Asians B) MD_2_ in Asians C) MD_1_ in Caucasians D) MD_2_ in Caucasians


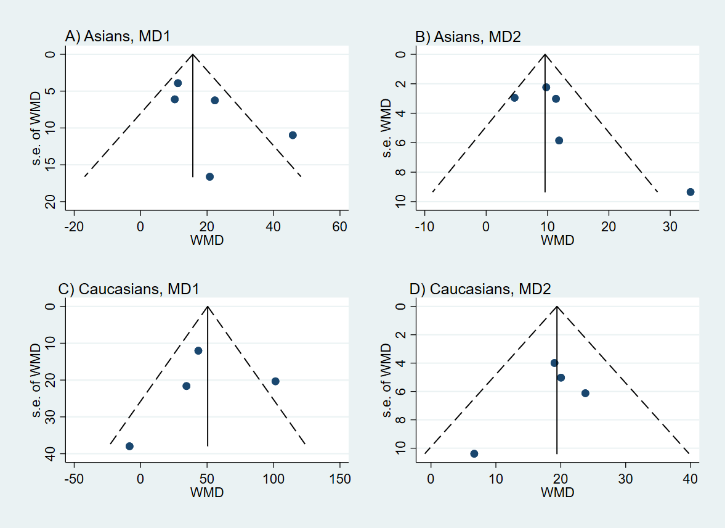


### Additional file 6.4.2. Funnel plots of rs1014290 on serum urate in Asians and Caucasians. A) MD_1_ in Asians B) MD_2_ in Asians C) MD_1_ in Caucasians D) MD_2_ in Caucasians


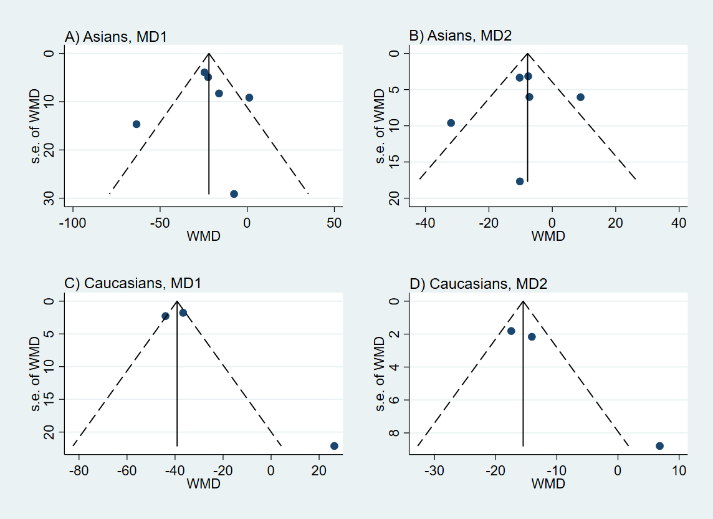


### Additional file 6.4.3. Funnel plots of rs6449213 on serum urate in Caucasians. A) MD_1_ in Caucasians B) MD_2_ in Caucasians


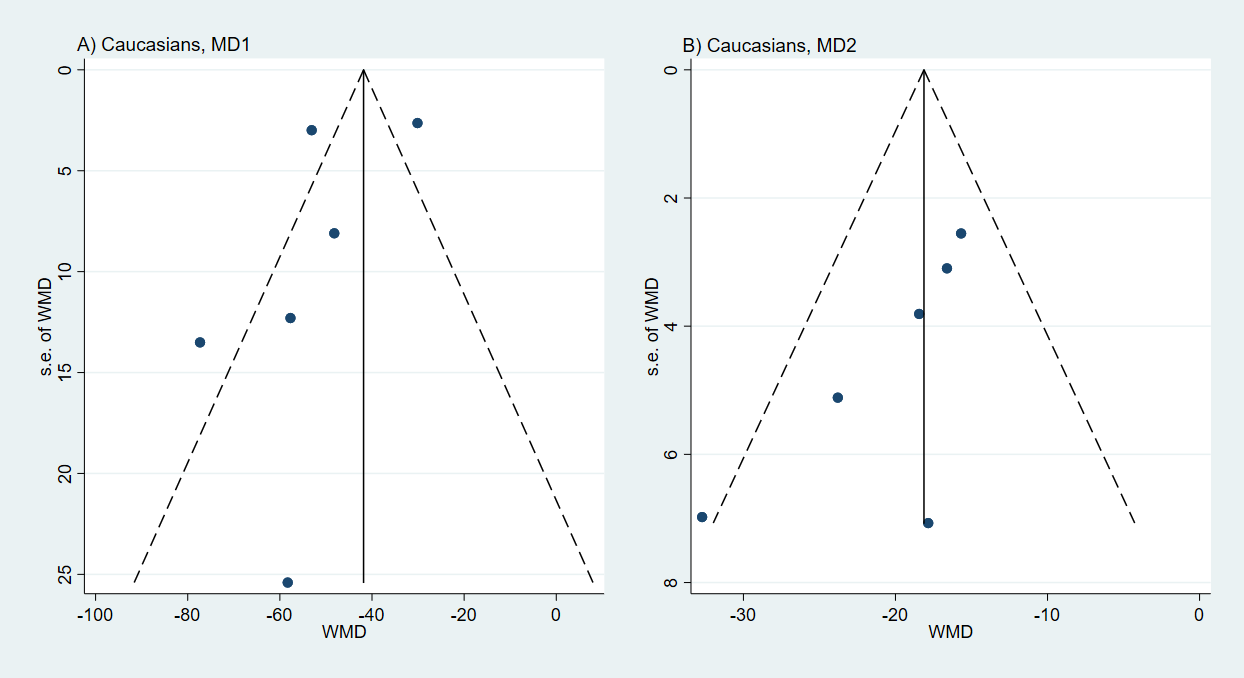


### Additional file 6.4.4. Funnel plots of rs7442295 on serum urate in Caucasians. A) MD_1_ in Caucasians B) MD_2_ in Caucasians


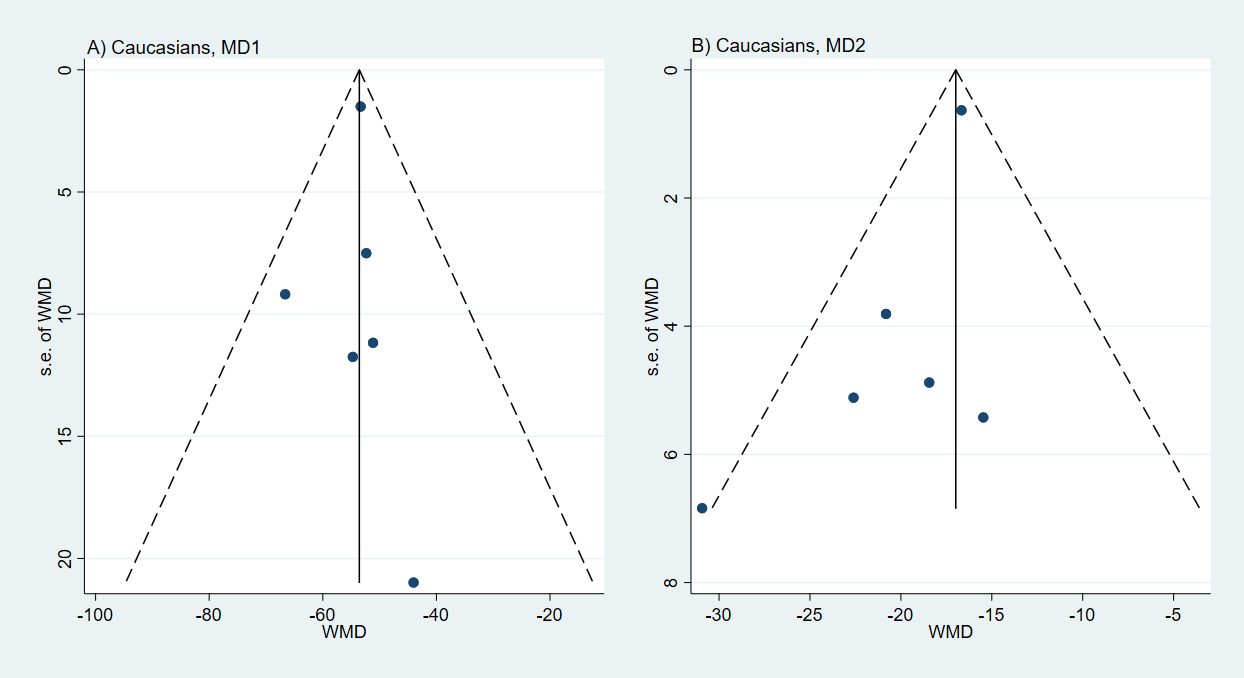


### Additional file 6.4.5. Funnel plots of rs6855911 on serum urate in Caucasians. A) MD_1_ in Caucasians B) MD_2_ in Caucasians


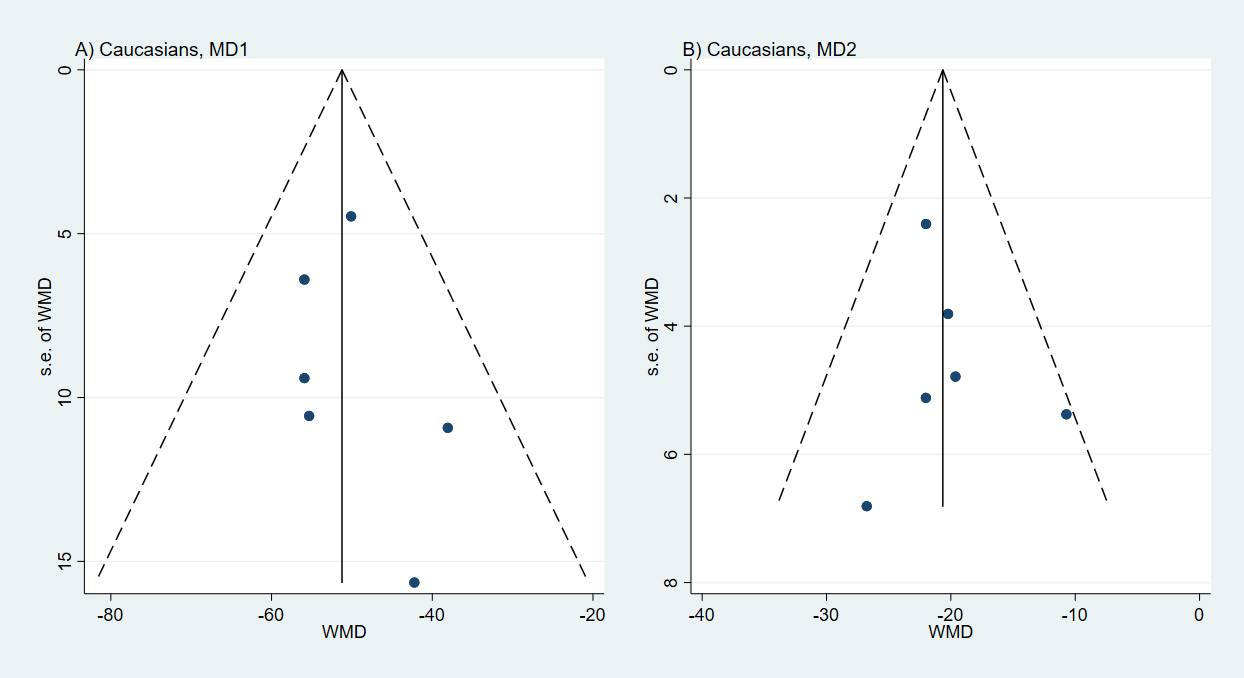


### Additional file 6.4.6. Funnel plots of rs734553 on serum urate in Caucasians. A) MD_1_ in Caucasians B) MD_2_ in Caucasians


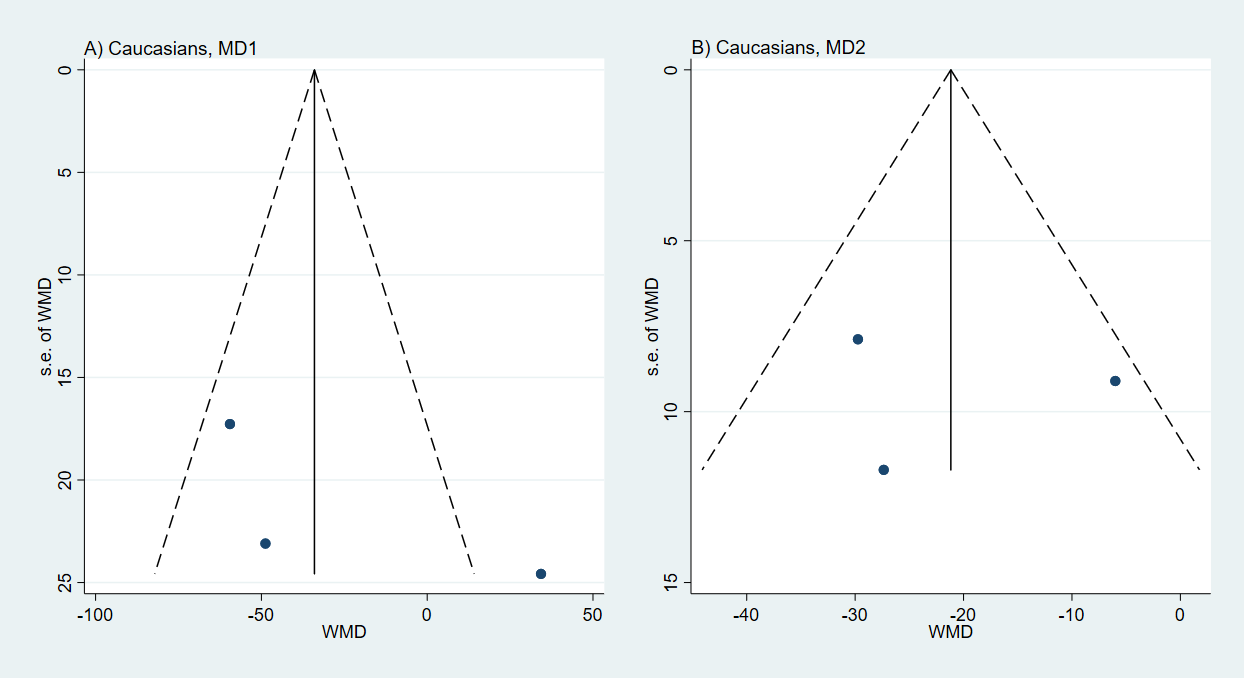

Supplement: Supplementary file 6 — Serum urate. 6.1. Data used for pooling mean difference of ABCG2 and SLC2A9 polymorphisms on serum urate. 6.2. Exploring source of heterogeneity for ABCG2 and SLC2A9 polymorphisms on serum urate. 6.3. Egger’s tests for ABCG2 and SLC2A9 polymorphisms on serum urate. 6.4. Funnel plots for ABCG2 and SLC2A9 polymorphisms on serum urate. (DOCX 412 kb) [file 12881_2020_1147_MOESM6_ESM.docx]
